# Post-sepsis chronic muscle weakness can be prevented by pharmacological protection of mitochondria

**DOI:** 10.1186/s10020-024-00982-w

**Published:** 2024-11-19

**Authors:** Meagan S. Kingren, Alexander R. Keeble, Alyson M. Galvan-Lara, Jodi M. Ogle, Zoltán Ungvári, Daret K. St Clair, Timothy A. Butterfield, Allison M. Owen, Christopher S. Fry, Samir P. Patel, Hiroshi Saito

**Affiliations:** 1 Department of Pharmacology and Nutritional Sciences, University of Kentucky, Lexington, KY USA; 2 Center for Muscle Biology, University of Kentucky, Lexington, KY USA; 3 Aging and Critical Care Research Laboratory, Department of Surgery, University of Kentucky, Lexington, KY USA; 4 Department of Physiology, University of Kentucky, Lexington, KY USA; 5 Department of Athletic Training and Clinical Nutrition, University of Kentucky, Lexington, KY USA; 6 Department of Rehabilitation Sciences, University of Kentucky, Lexington, KY USA; 7 Department of Toxicology and Cancer Biology, University of Kentucky, Lexington, KY USA; 8 Markey Cancer Center, University of Kentucky, Lexington, KY USA; 9 Spinal Cord and Brain Injury Research Center, University of Kentucky, Lexington, KY USA; 10 Vascular Cognitive Impairment, Neurodegeneration and Healthy Brain Aging Program, Department of Neurosurgery, University of Oklahoma Health Sciences Center, Oklahoma City, OK USA; 11 Oklahoma Center for Geroscience and Healthy Brain Aging, University of Oklahoma Health Sciences Center, Oklahoma City, OK USA; 12 Department of Health Promotion Sciences, College of Public Health, University of Oklahoma Health Sciences Center, Oklahoma City, OK USA; 13 Doctoral College/Department of Public Health, International Training Program in Geroscience, Semmelweis University, Budapest, Hungary

**Keywords:** Post-sepsis syndrome, Muscle weakness, Mitochondrial myopathy, Critical care illness

## Abstract

**Background:**

Sepsis, mainly caused by bacterial infections, is the leading cause of in-patient hospitalizations. After discharge, most sepsis survivors suffer from long-term medical complications, particularly chronic skeletal muscle weakness. To investigate this medical condition in detail, we previously developed a murine severe sepsis-survival model that exhibits long-term post-sepsis skeletal muscle weakness. While mitochondrial abnormalities were present in the skeletal muscle of the sepsis surviving mice, the relationship between abnormal mitochondria and muscle weakness remained unclear. Herein, we aimed to investigate whether mitochondrial abnormalities have a causal role in chronic post-sepsis muscle weakness and could thereby serve as a therapeutic target.

**Methods:**

Experimental polymicrobial abdominal sepsis was induced in 16-18 months old male and female mice using cecal slurry injection with subsequent antibiotic and fluid resuscitation. To evaluate the pathological roles of mitochondrial abnormalities in post-sepsis skeletal muscle weakness, we utilized a transgenic mouse strain overexpressing the mitochondria-specific antioxidant enzyme manganese superoxide dismutase (MnSOD). Following sepsis development in C57BL/6 mice, we evaluated the effect of the mitochondria-targeting synthetic tetrapeptide SS-31 in protecting mitochondria from sepsis-induced damage and preventing skeletal muscle weakness development. In vivo and in vitro techniques were leveraged to assess muscle function at multiple timepoints throughout sepsis development and resolution. Histological and biochemical analyses including bulk mRNA sequencing were used to detect molecular changes in the muscle during and after sepsis

**Results:**

Our time course study revealed that post sepsis skeletal muscle weakness develops progressively after the resolution of acute sepsis and in parallel with the accumulation of mitochondrial abnormalities and changes in the mitochondria-related gene expression profile. Transgenic mice overexpressing MnSOD were protected from mitochondrial abnormalities and muscle weakness following sepsis. Further, pharmacological protection of mitochondria utilizing SS-31 during sepsis effectively prevented the later development of muscle weakness.

**Conclusions:**

Our study revealed that the accumulation of mitochondrial abnormalities is the major cause of post-sepsis skeletal muscle weakness. Pharmacological protection of mitochondria during acute sepsis is a potential clinical treatment strategy to prevent post-sepsis muscle weakness.

**Supplementary Information:**

The online version contains supplementary material available at 10.1186/s10020-024-00982-w.

## Background

While the global sepsis mortality rate has decreased over the last 20 years, sepsis incidence steadily risen (Kingren et al. 2021; Rudd et al. 2020; Angus et al. 2001; McDermott 2018; Liang et al. 2017) in Western countries, creating nearly 1.9 million new sepsis survivors annually in the US alone (Kingren et al. 2021; McDermott U.S. Hospitals 2018; Liang et al. 2017; Elixhauser et al. 2009; Martin et al. 2006). A majority of survivors suffer from debilitating complications collectively termed Post-Sepsis Syndrome, the symptoms of which include cognitive deficits (Wang et al. 2021; Li et al. 2022), impaired muscle function (Owen et al. 2019; Schefold et al. 2010; Callahan and Supinski 2009), and persistent fatigue (Slikke et al. 2023) among others. Muscle weakness affects 70–100% of severe sepsis survivors and is associated with an elevated 5-year mortality rate (Schefold et al. 2010; Khan et al. 2006; Witt et al. 1991; Tennila et al. 2000; Piva et al. 2019; Mankowski et al. 2021). While muscle wasting during acute sepsis is commonly thought to be a significant cause of this prolonged weakness (Schefold et al. 2010; Yoshihara et al. 2023), deficits remain after atrophy resolution in both human subjects (Santos, et al. 2016) and our murine model of sepsis (Owen et al. 2019). Accordingly, the precise mechanisms underlying post-sepsis skeletal muscle weakness remain unclear, with causes other than atrophy alone likely being major contributors to chronic weakness. This knowledge gap contributes to the lack of effective therapies to restore function following sepsis.

Our previous research using a murine model of severe sepsis survivors indicates that mice suffering long-term muscle weakness after complete restoration of muscle mass have profound mitochondrial abnormalities (Owen et al. 2019). However, both the timing of these mitochondrial abnormalities and whether they are causally linked to decreased skeletal muscle function following sepsis remain unknown. Herein, we conducted a study to test our hypothesis that mitochondrial abnormalities are the major cause of post-sepsis skeletal muscle weakness and that such weakness can be effectively prevented by targeting mitochondria therapeutically.

Utilizing a time-course approach, we first show that both mitochondrial abnormalities and post-sepsis weakness increase gradually from the acute sepsis phase (≤ 4 days after sepsis induction) to the post-sepsis chronic phase (≥ 2 weeks post sepsis induction). We also show that the muscle weakness persists even after acute muscle wasting is completely resolved. Then we provide evidence for mitochondrial abnormalities as major *causes* of post-sepsis muscular dysfunction by utilizing a transgenic strain of mice overexpressing the mitochondria-localizing antioxidant enzyme manganese superoxide dismutase (MnSOD or SOD2). Both post-sepsis mitochondrial abnormalities and skeletal muscle weakness were well protected in mice overexpressing MnSOD, whereas wildtype mice demonstrated significant functional deficits and increased mitochondrial abnormalities. Further, we found that the mitochondria-protecting pharmacologic synthetic tetrapeptide SS-31 (Zhao et al. 2005) can significantly protect against the development of post-sepsis complications, even when administered after severe illness development. Accordingly, we conclude that mitochondrial protection during acute sepsis is a viable preventive therapy in limiting mitochondrial abnormalities and subsequent debilitating post-sepsis skeletal muscle weakness.

## Methods

### Animals and husbandry

Late middle-aged male C57BL/6 mice (age 14–16 months) were obtained from the National Institute on Aging and acclimated for at least two weeks in the University of Kentucky Division of Laboratory Animal Resources (DLAR) prior to the beginning of experimentation at age 16–18 months. Young adult male C57BL/6 (age 4 months) were obtained from The Jackson Laboratory to prepare cecal slurry stock solutions. Transgenic mice overexpressing human MnSOD (Yen et al. 1996) (MnSOD-TG) and their littermate wild-type control mice (MnSOD-WT) were bred on a C57BL/6 background, and the colony was maintained in the University of Kentucky Division of Laboratory Animal Resources. Male and female MnSOD-TG and WT mice were 16–18 months old when used in experimental sepsis procedures. Genotyping was completed after weaning utilizing DNA from ear punches. Mice were housed 3–5 animals per cage in pressurized intra-ventilated cages on a 14/10 h light–dark cycle with ad libitum access to chow and water. Both temperature (21–23°C) and humidity (30–70%) were kept constant throughout the study.

All animal experiments were conducted according to procedures described in our Animal Use Protocol #2009–0541 which is approved by the Institutional Animal Care and Use Committee at the University of Kentucky. All procedures were in accordance with the National Institute of Health’s guidelines for ethical treatment.

### Sepsis induction and resuscitation

For experimental sepsis induction by cecal slurry (CS) injection, all mice (C57BL/6 and transgenic WT/TG mice) were aged to 16–18 months of age (equivalent to ~ 50–55 human years old (Preau et al. 2021)) and randomized to treatment group based on body mass. The CS stock solution was prepared as we described previously (Steele et al. 2017). Briefly, stools were collected from the cecum of C57BL/6 mice, dissolved in 10% glycerol saline solution (1-mL for every 100 mg of stool), strained through a series of screen mesh, and stored in multiple cryovials at -80 °C. A single batch of CS stock (Lot number CS-191206) was used throughout the study, except transgenic mouse experiments which used CS-190705. After preparation of CS stock, we determined the minimum lethal dose in which CS injection would prove 100% fatal if not resuscitated with antibiotics and fluids. Upon selection of the correct cecal slurry dose, the CS stock was thawed in a water bath at 37 °C and injected into mice intraperitoneally (*i.p.*) using a 1 mL syringe with a 25-guage needle. Six hours after sepsis induction, body temperatures were monitored with a rectal temperature probe (RET-3 Physiotemp Instruments, Clifton NJ attached to a Thermocouple Meter Model 20250–91, Davis Instruments, Hayward, CA). At the 12 h mark, temperatures were recorded once again and again every 12 h thereafter for at least 5 days or until mice returned to a normal body temperature (37 °C). Body weight was also monitored daily. Late-stage resuscitation with sterile saline (0.7 mL, subcutaneous injection) and antibiotics (imipenem, IPM reconstituted in sterile physiological saline; 1.5 mg/injection (0.3 mL)) was initiated at 12 h after sepsis induction, when animals are profoundly sick, and continued 2× daily for 5 days for a total of 9 injections with saline and antibiotics, each, as we described (Owen et al. 2019; Steele et al. 2017).

For euthanasia, mice were anesthetized via inhalation of 2.5% isoflurane and a laparotomy was performed to open the abdominal cavity. The IVC was cut to ensure exsanguination. Mice were euthanized on days 0, 3, and 14 to assess when mitochondrial damage develops. Non-sepsis controls were euthanized on day 0 (pre-sepsis), while septic mice were euthanized at 4 (acute sepsis), and 14 days for the time-course study portion of this study. MnSOD WT and TG mice were euthanized either on day 0 (non-sepsis controls) or day 21 (sepsis survivors). For animals treated with SS-31, animals were euthanized on day 0 (non-sepsis controls) or 28 (sepsis survivors). In mice undergoing in vivo skeletal muscle function testing, mice were observed through day 70. Upon euthanasia, hindlimb muscles were collected. The left tibialis anterior (TA) was covered in optimal cutting temperature (O.C.T.) compound (Tissue-Tek, #4583) at resting length and frozen in liquid nitrogen cooled isopentane for storage at −80 °C. The right TA and gastrocnemius muscles were snap frozen in liquid nitrogen and stored at −80°C. A small portion of the distal end of the extensor digitorum longus (EDL) was removed and stored in EM buffer at 4°C. The wet tissue weight of the spleen was recorded to assess sepsis severity through the degree of splenomegaly as previously described (Owen et al. 2019 . ). Mice that did not demonstrate splenomegaly (> 100 mg wet weight), severe hypothermia (< 30°C at 6 or 12 h), and significant weight loss during the first two weeks were not considered to be severely septic and thus eliminated prior to analysis and further experimentation.

### Treatment with SS-31

SS-31 was obtained from GeneScript. SS-31 was reconstituted using 40 mL of sterile saline to achieve a concentration of 10 mg/kg of body weight per mouse (avg body weight 35 g). Following sepsis induction, mice were randomized to SS-31/sham treatment groups based on 6-h body temperature measurements to ensure equivalent sepsis severity. SS-31 was incorporated into the resuscitation protocol described above wherein 0.7 mL of SS-31-enriched-saline was injected subcutaneously every 12 h for 5 days in tandem with *i.p.* injection of 0.3 mL of broad-spectrum antibiotics. Control mice received standard saline as a vehicle control to ensure all mice received the same amount of fluid following sepsis. Non-sepsis control mice received the same volume of saline/SS-31-enriched-saline.

### In vivo muscle function testing

Using an Aurora Scientific 1300A: 3-in-1 Whole Animal System-Mouse apparatus, we assessed plantar flexion function as previously described (Englund, et al. 2021). After ensuring the 300D-300C-FP motor with the ankle flexion apparatus was attached to the 809C animal platform and the platform was heated to 37 °C using an attached water pump, mice were anesthetized using 2.5% isoflurane with 400 mL/min oxygen. The right leg was then shaved to remove fur and the mouse was placed in a supine position on the platform with its head in the nosecone to continue isoflurane administration. The knee was clamped at 90°, and the foot was placed on the force plate, with the tibiofibular at a 90° angle relative to the footplate. Then, a piece of tape was placed to secure the foot to prevent any compensatory movement. Two electrodes deliver the stimulus and must be placed subcutaneously 1–2 mm apart probing the peroneal nerve. To test for optimal placement, the probes were placed distal to the knee joint and then a single 350 ms stimulus at 50 mA was delivered. Once optimal placement was obtained by moving one probe, testing torque, and then moving the other probe, optimal current was determined. Current output was initially set at 50 mA and twitch torque was recorded with the baseline subtracted. Current was then increased or decreased by 10 mA until the maximum twitch torque was recorded. This lowest current at which maximum twitch torque was achieved was recorded and utilized throughout the rest of the torque-frequency experiment.

To assess the torque-frequency relationship and determine peak isometric torque, the pre-programmed plantar-flexion protocol was used to deliver 350 ms stimuli in increasing frequency: 10 Hz, 40 Hz, 120 Hz, 150 Hz, 180 Hz, and 200 Hz with a rest period between contractions of 120 s. Following completion of testing, the electrodes were removed from the mouse and its knee was unclamped. The isoflurane was turned off and the mouse was placed back in its cage and monitored until it regained consciousness.

Function testing was performed in mice prior to sepsis induction to serve as a baseline. On day 3 after sepsis induction, mice were subjected to a second round of testing. The last round of function testing was carried out on day 14 after which mice were anesthetized. Mice that died as a result of sepsis were excluded from analysis, as they were not considered sepsis survivors. Repeat testing in the same mice throughout sepsis pathophysiology enabled true comparison of muscle weakness over time and the ability to see if function ever returns to baseline/pre-sepsis status.

### In vitro muscle function testing

Immediately following euthanasia, the left hindlimb was skinned and placed in oxygenated (95%O_2_-5% CO_2_) Krebs–Ringer solution. Due to our previous research indicating that fast twitch muscles are particularly susceptible to the effects of sepsis-induced muscle weakness, we utilized the EDL for most of these experiments. The muscle bath was continuously oxygenated while the EDL was dissected under a microscope and sutures were secured in the tendons in close proximity to the in the proximal and distal myotendinous junctions using braided silk (4–0). After freeing the muscle from the leg, it was mounted with the distal end attached to a hook secured to a moveable stage and the proximal end attached to the lever arm of an ASI 300C-LR Aurora Scientific signal transducer system. The platinum electrodes were moved into place around the muscle and the continuously oxygenated muscle bath was raised to allow the EDL to acclimate for five minutes at 25°C. Afterwards, optimal length (Lo) or the length at which the EDL produced peak maximal twitch torque, was determined by incrementally adjusting the EDL length between twitches until the maximum twitch force was obtained. Once Lo was obtained, the stage was locked, and the EDL fascicle length was measured using digital calipers.

While at Lo, the muscle was subjected to a series of stimulations (500 ms in length) to obtain the force-frequency relationship. Frequencies used were 1, 15, 30, 50, 80, 150, 250, and 300 Hz. Between each isometric contraction, there was a 60s rest followed by a 500 ms train at 300 Hz followed by another 60 s rest, followed by the next 500 ms train at a new frequency. Following conclusion of the protocol, the muscle was once again measured with digital calipers to ensure there were no changes in muscle length during the protocol. The EDL was then carefully removed from the apparatus, and the sutures and remaining tendon material were removed to obtain the muscle wet weight. The EDL was then placed in a cryovial and frozen in liquid nitrogen and stored at −80°C. peak isometric force was then normalized to the calculated physiological cross-sectional area (PCSA), as previously described (Owen et al. 2019).

### RNA sequencing

Upon euthanasia and hindlimb muscle dissection, the right tibialis anterior was flash frozen in liquid nitrogen and stored at -80°C. Samples were placed in 1.5 mL tubes with 700uL of TRIzol and a metal bead for homogenization using a TissueLyser. RNA was then isolated using an RNA isolation kit (PureLink RNA Mini Kit; cat no. 12183018A; Invitrogen, Carlsbad, CA, USA) as per manufacturer’s instructions. DNase treatment was conducted on-column using DNase (DNA-free Kit DNase Treatment & Removal; Invitrogen, Carlsbad, CA, USA) as per manufacturer’s instructions. RNA concentration was assessed, and isolated RNA was sent to Novogene for library construction, sequencing, and preliminary bioinformatic analyses.

Partek Flow was utilized to align the raw FASTQ files using the STAR aligner. The aligned counts were subjected to differential analysis using DESeq2 in R 4.2.2 + . The data was then normalized and DEGs were identified by a P-adj value < 0.05. PCA plots and heatmaps were then generated in R packages available through Github. Following identification of DEGs, pathway over-representation analysis was completed using gProfiler (Reimand et al. 2007) and the top up- and down-regulated pathways were noted. All reported GO: Cellular Component, GO: Molecular Function, and GO: Biological Function pathways reported were calculated to include at least 3 genes implicated in the altered pathways and were filtered utilizing a Benjamini–Hochberg value of 0.05 to exclude false-positives.

### Histological analyses

Upon euthanasia and hindlimb muscle dissection, the left tibialis anterior was embedded in a layer of optimal cutting temperature (OCT) (Tissue-Tek, #4583). Pinned to a foil-covered cork at resting length, the muscle was frozen via submersion in isopentane prechilled by liquid nitrogen. Following freezing, the cork was placed on dry ice where the OCT embedded TA was removed and placed into a prechilled cryovial for storage at −80 °C until sectioning. Frozen tissue was sectioned using a cryostat (HM525-NX, Thermo Fisher Scientific, Waltham, MA, USA) in 7um thick sections, air dried on slides, and subsequently stored at −20°C for short-term storage, or −80°C for long-term storage.

For SDH (succinate dehydrogenase) staining, thawed skeletal muscle cross-sections were incubated in 100 mM sodium succinate salt and 1.2 mM NBT in 0.2 M PBS for 1 h in an agitated water bath at 37 °C. Tissues were then rinsed with DI water, washed in a series of acetone solutions (30% for 1 min, 60% for 1 min, 30% again for 1 min, all at RT, and finally 100% acetone at -20° for 20 min), rinsed again, and washed with 1 × PBS twice for 3 min. Sections were then mounted with Vectashield mounting medium or incubated with primary antibody overnight for fiber-typing. NADH (nicotinamide adenine dinucleotide) staining followed standard procedures. Thawed sections were incubated in 2.4 mM NADH and NBT in 0.5 M Tris buffer for 30 min on a plate rocker at 37 °C. Prior to applying the NADH solution, sections were circled with a PAP sectioning pen to enable better coverage of the solution per individual tissue section. Sections were then rinsed, fixed, and mounted as described above for SDH staining. After imaging, quantification was carried out using Aperio ImageScope software looking at the mean staining intensity of each individual fiber. This was then averaged for each section and compared across groups.

To assess fiber type distribution and cross-sectional area of TA muscles, sections were dried and rehydrated with PBS for three minutes prior to staining fiber-type-specific isoforms of MyHC unless coming immediately from SDH staining. Sections were incubated rocking at 4 °C overnight in primary antibodies from Developmental Studies Hybridoma Bank (DSHB, Iowa City, IA, USA): type I (1:100; BA.D5-C; IgG2b), type IIa (1:50; SC.71; IgG1), type IIx (served as diluent, 6H1; IgM), with laminin for fiber borders (1:100; Sigma, Cat#L9393) and type IIb fibers remaining unstained. Slides were then washed 3 times for 3 min each time in PBS. Secondary antibodies from Invitrogen were diluted in PBS and incubated at room temperature for 1 h: anti-mouse IgG2b, AF647 (1:250; #A21242), anti-mouse IgG1, AF488 (1:250; #A21121), anti-mouse IgM, AF555 (1:250; #A21426), and anti-rabbit IgG, AF350 (1:250, #A21068). Following 3 PBS washes for 5 min each, sections were incubated in Steptavidin-AMCA (1:200) in 1× PBS for 1 h at room temperature and washed with PBS again. Slides were then mounted with Vectashield mounting media (Vector, cat#H-1000). Entire muscle cross-section images were then captured with an upright microscope at 100–200 × magnification (AxioImager M1, Zeiss, Oberkochen, Germany). MyoVision 2.0 (Wen et al. 2018) was utilized to quantify fibertype and assess cross-sectional area per each myofiber based on the intensity within the Cy5, GFP, and Texas Red filters for type I, IIa, and IIx fibers respectively, with type IIb fibers remaining unstained and myofiber borders being marked by laminin which showed blue.

### Transmission electron microscopy (TEM)

Upon euthanasia, a small section of the distal EDL was placed in EM buffer (2% paraformaldehyde, 2.5% glutaraldehyde in 0.1 M cacodylate buffer, pH 7.4; Electron Microscopy Sciences; Hatfield, PA, USA). Electron microscopy imaging was performed in the HMS electron microscopy facility at Harvard University. There, tissues were post-fixed and dehydrated as described previously (Owen et al. 2019). After overnight infilatration in a 1:1 mixutre of propylene oxide and TAAB Epon (Marivac Ltd., St. Laurent, Canada), sections were viewed under the Philips Technai BioTwin Spirit Electron Microscope (FEI, Hillsboro, OR, USA). At least 10 views at both 5,000x and 15,000x were captured for each sample in a blinded manner. Samples were then quantified using the cell counter feature on ImageJ. Ten separate areas of each sample were imaged under two different magnifications (5,000x and 15,000x), and mitochondria were classified as healthy or aberrant.

### Protein isolation and Western blotting

Upon euthanasia and hindlimb muscle dissection, the gastrocnemius muscles were placed in cryovials and flash frozen in liquid nitrogen for storage at −80 °C. Muscles were isolated individually in 700 uL of protein isolation buffer in round bottom 2 mL Eppendorf tubes with 2 metal beads. Samples were then added and a TissueLyser LT was used to isolate protein. Homogenate was then collected, added to a new 2 mL Eppendorf tube, and heated in a water bath at 80 °C for five minutes and subsequently centrifuged (12,000 g for 10 min at room temperature). Samples were then aliquoted for quantification and storage at −80 °C. Protein concentration was assessed using the Bio-Rad *RC DC* (cat# 500–0120) assay as per the manufacturer’s instructions.

Samples (20 g) were separated by SDS-PAGE electrophoresis (Mini PROTEAN TGX Stain-Free Gels, cat # 4568096, BioRad) using pre-cast stain-free gradient (4–20%) gels, whereafter total protein was measured for later normalization via stain-free imaging. Samples were then transferred onto PVDF membranes (Trans-Blot Turbo Pack, cat # 17004157) (except for blots where 3-NT was the primary antibody, in which case nitrocellulose membranes were used; Trans-Blot Turbo Transfer Pack, cat # 1704158). Blots were blocked in 5% blotting grade blocker (BioRad cat # 170–6404) for 1 to 2 h at room temperature. Blots were incubated overnight at 4 °C with primary antibody (3-NT ab 61392 1:3000; 4-HNE ab46545 1:3000; OXPHOS Rodent Antibody Cocktail STN-19467, 1:250). Blots were then washed and incubated with secondary antibody for 1 h. The membranes were then treated with Clarity Western ECL substrate (BioRad cat# 170–5060) and detected by chemiluminescence. Band volume was detected using Image Lab software and normalized to total protein.

### Overall statistical analyses

Previous power analyses were conducted by Dr. Arnold Stromberg at the University of Kentucky and indicated that 6 animals per group were sufficient to have a well-powered study, with 4 being sufficient for the SS-31 studies. Student’s T-tests were applied when comparing two means from independent samples, and a one-way analysis of variance (ANOVA) was used when comparing the means of three or more data sets. Two-way ANOVAs were used to compare the effect of a treatment over time across different groups. Appropriate post-hoc analyses were conducted as needed. To align raw FASTQ files for sequencing, the STAR aligner was used, and quality control was checked, with reads being trimmed when necessary. Counts were then processed for differential analyses using DESeq2 and subsequently normalized. P-adj values of 0.05 or less were considered significant for subsequent over-representation analyses as described previously. In general, P-values of less than 0.05 were considered statistically significant. Analyses were carried out in GraphPad Prism and R 4.2.2 + .

## Results

### Skeletal muscle function decreases progressively after sepsis

**Fig. 1:**
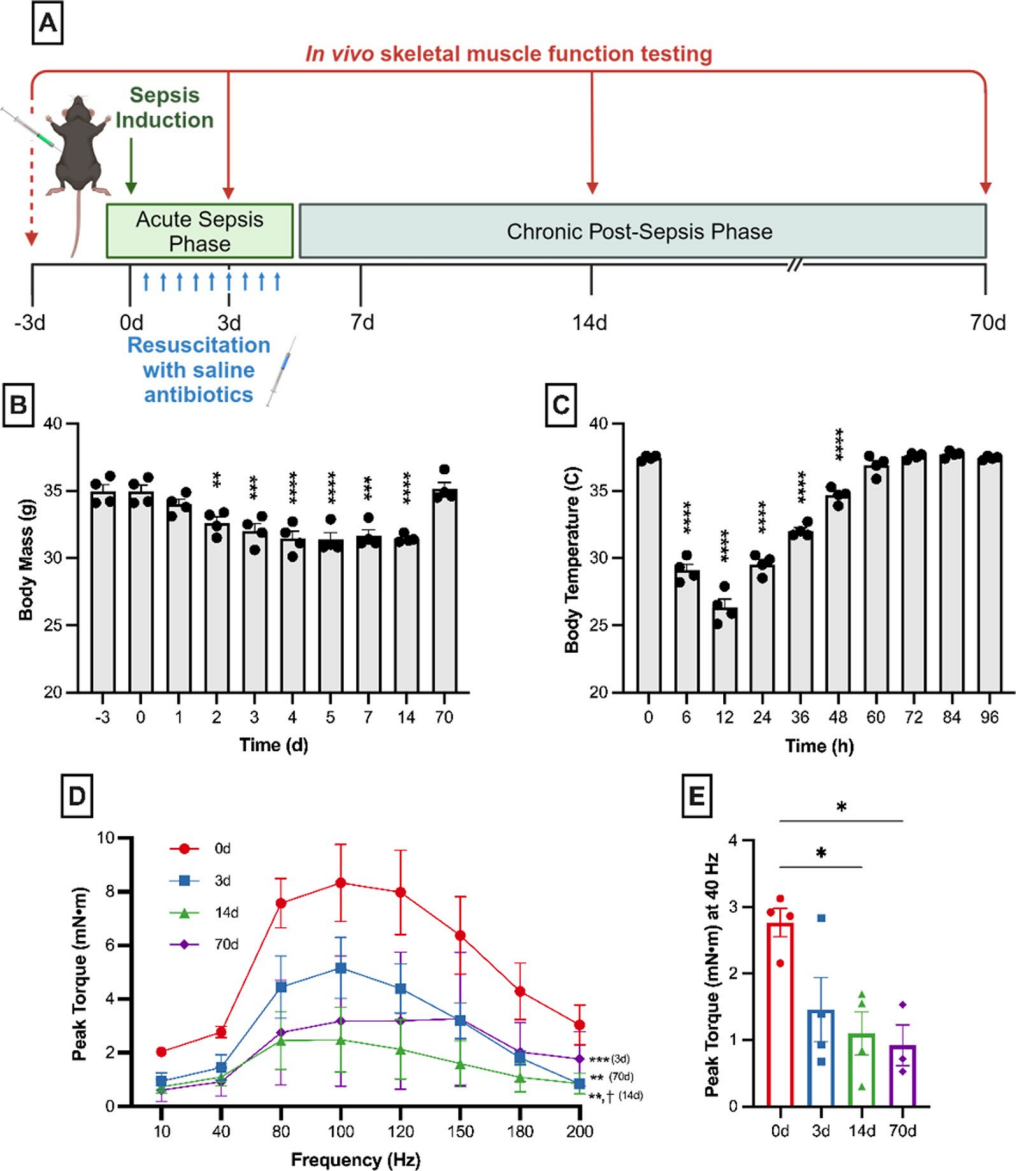
Skeletal muscle weakness develops progressively after sepsis resolution. **A** Late middle-aged C57BL/6 mice were subjected to severe sepsis followed by resuscitation with antibiotics and saline beginning 12 h after sepsis induction. Sepsis surviving mice (n = 4) suffered severe acute sepsis illness with significant decreases in body mass **(B)** and temperature **(C)**. **D** Effect of sepsis on muscle function was examined by testing in vivo plantar flexion on the same four mice prior to sepsis induction, during acute illness (3d), and following recovery from sepsis (14d and 70d). Post-sepsis skeletal muscle weakness was evident by day 3 and persisted through at least day 70. Notably, skeletal muscle weakness worsened from day 3 to day 14, did not recover by day 70, and was still significantly weaker than pre-sepsis levels. **E** Analysis of plantar flexion at a physiologically relevant stimulus frequency (40 Hz) showed significantly reduced muscle function in the chronic post-sepsis phase. Data was expressed as mean ± SEM. Difference from baseline was expressed as *P < 0.05, **P < 0.01, ***P < 0.001, and ****P < 0.0001 (**B**-**E**), while difference from day 3 was denoted with ꝉ *P* < 0.05 and ꝉꝉ *P* < 0.01 (**D**-**E** only)

To determine how sepsis alters skeletal muscle function progressively over time, we utilized an in vivo plantar flexion function testing protocol. First, baseline measurements were taken 3 days before sepsis induction (day -3). After abdominal sepsis was induced utilizing the cecal slurry injection model of sepsis (on day 0) (Owen et al. 2019; Steele et al. 2017; Starr et al. 2014), muscle function tastings were repeated during acute sepsis (days 3–4), and during chronic post-sepsis phase (on day 14 and day 70) (outlined Fig. 1A).

Within 6 h after cecal slurry injection, mice became severely hypothermic, and average body temperature remained significantly decreased through 24 h. Mice received resuscitation with antibiotics and saline beginning 2 × daily for five days beginning 12 h after cecal slurry injection. Mice were again subjected to muscle function testing during acute sepsis (days 3–4), and peak isometric tetanic plantar flexor torque had decreased 38.5% from baseline function. After complete recovery from acute sepsis [confirmed by a return to normal body temperature, increasing body weight (Fig. 1B–C), and resolution of systemic inflammation as noted by plasma cytokines (Owen et al. 2019), muscle function was tested again on day 14. Peak torque had further decreased 49.8% from day 3 to day 14. In other words, on day 14, sepsis survivors could only produce 30.9% of their baseline function at peak frequency (equivalent of a 69.1% decrease from day 0 function). Mice were then evaluated on day 70, or 10 weeks after sepsis induction. Function deficits were still persistent at day 70 with no discernable differences from day 3 or 14 function (Fig. 1D). When compared to baseline function, day 70 function was 56.8% lower than pre-sepsis function, meaning mice were only capable of 43.2% of baseline function at peak frequency. Further, when looking at a more physiologically-relevant stimulus frequency (40 Hz), torque was significantly reduced at 14 and 70 days (Fig. 1E). Thus, it is clear skeletal muscle weakness develops gradually after acute sepsis and persists for months after sepsis recovery.

### Transcriptional changes occur throughout sepsis progression.

**Fig. 2:**
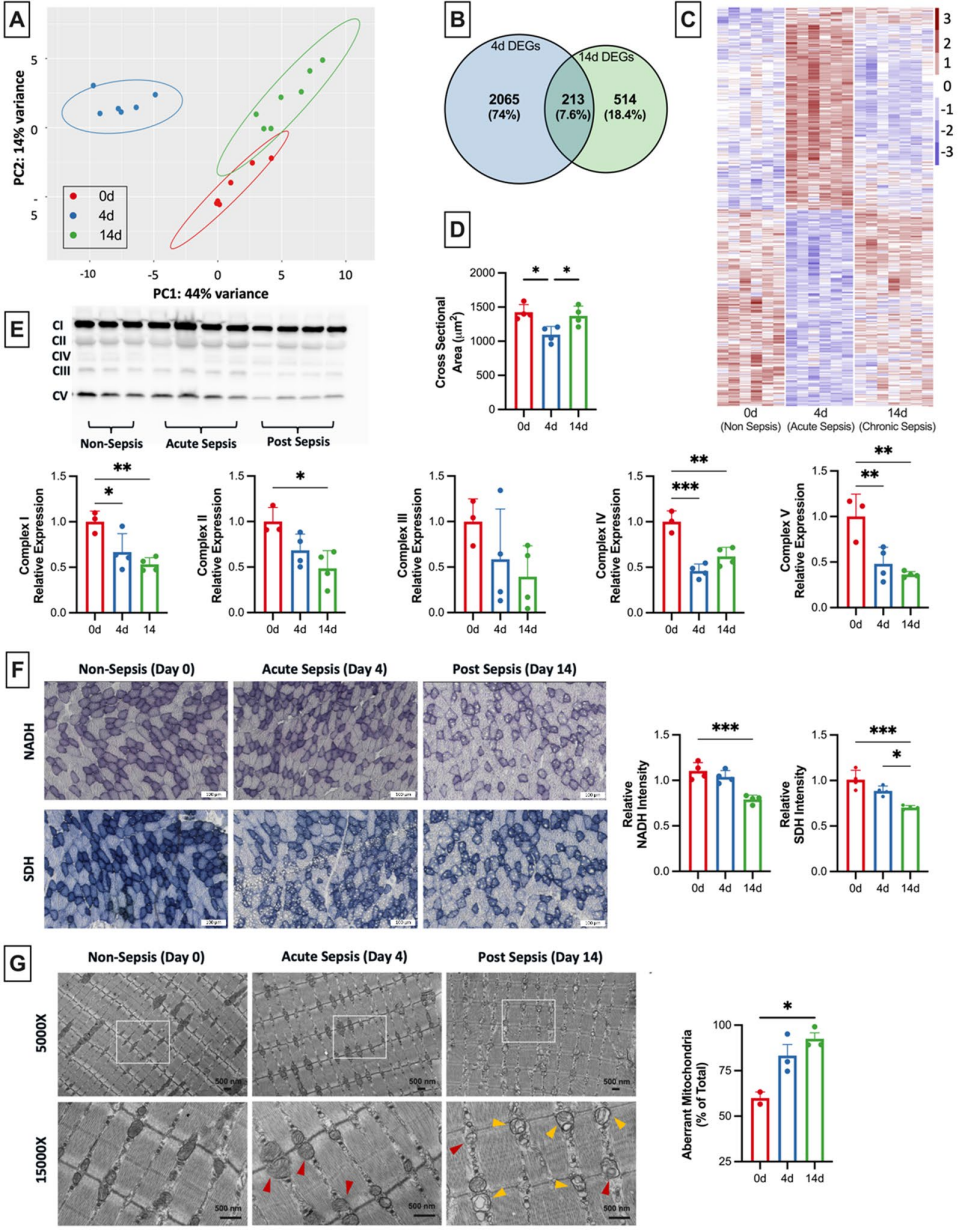
Skeletal muscle mitochondrial abnormalities develop progressively. Bulk mRNA sequencing was conducted on TA muscles from mice prior to sepsis (0d), during acute sepsis (4d) and after recovery during the chronic sepsis phase (14d). **A** Samples clustered based on sepsis timepoint (n = 6–7) as shown by PCA. **B** Out of the 2,278 DEGs at day 4, 213 of these remained altered on day 14, at which point an additional 514 DEGs emerged. Venn diagram depicts the number of DEGs only altered at day 4 (2065; 74%) and day 14 (514; 18.4%) on the left and right, respectively, with the DEGs altered at both timepoints shown in the middle (213; 7.6%). **C** A heatmap of the DEGs is shown. **D** Cross sectional area was reduced at day 4 and recovered by day 14, demonstrating that atrophy in the acute phase is recovered by the chronic post-sepsis phase **(E)** Western blot analyses demonstrated decreased expression of complexes I, IV, and V by day 4 that remained depressed at day 14. **F** In situ enzyme staining for complex I (NADH) and complex II (SDH) on TA muscle sections revealed intensity was significantly decreased by day 14, but not as early as day 4 during acute sepsis. **G** TEM was used to visualize changes in mitochondrial ultrastructure on in EDL samples from days 0, 3, and 14 (n = 3 each). Yellow and red arrow heads indicate examples of mitochondria with ruptured mitochondrial membranes and organelles with disrupted cristae/centralization into a vacuole-like structure, respectively. White squares in 5,000 × images indicate the area of 15,000 × images. Aberrant mitochondria accumulated progressively after sepsis, with a majority of the organelles demonstrating altered structure by day 14. Data is presented as mean ± SEM. Significant differences are compared to baseline where *P < 0.05, **P < 0.01, and ***P < 0.001

Noting that post-sepsis skeletal muscle weakness occurs progressively after sepsis, we induced sepsis in a second cohort of mice to evaluate molecular changes throughout sepsis pathogenesis. For this study, animals were euthanized on day 0 (before sepsis) and 4 and 14 days after sepsis induction, and hindlimb muscles were collected for analyses. Development of severe sepsis was evaluated in each animal by confirmation of significant hypothermia during acute sepsis and, upon euthanasia, splenomegaly, which is a marker of severe infection (Supplementary Fig. 1). Bulk RNA sequencing was performed on tibialis anterior (TA) hindlimb muscles. Principal component analysis (PCA) revealed three distinct clusters based on sepsis status (day 0, 4, or 14; Fig. 2A). Out of the 2318 differentially expressed genes (DEGs; adjusted-p-value ≤ 0.05) during acute sepsis (day 4), 1,160 transcripts were downregulated while 1158 were upregulated. A majority of these DEGs returned to normal levels by day 14, while 213 DEGs still remained altered. Another 514 genes were altered between days 4 and 14 to total 727 DEGs on day 14 as compared to day 0 (359 downregulated and 368 upregulated) (Fig. 2B–C).

We conducted separate pathway analyses for timepoints day 4 and day 14 using gProfiler. This analysis revealed significant alterations in pathways related to inflammation, immune regulation, oxidative stress, and skeletal muscle abnormalities during the acute phase of sepsis, as detailed in Supplementary Fig. 2. Subsequently, we focused on the 2,105 DEGs that showed normalization by day 14. A secondary pathway analysis of these genes indicated a resolution of most acute-phase abnormalities related to inflammation, oxidative stress, and immune regulation by day 14. In contrast, GO terms associated with mitochondrial dysfunction and skeletal muscle abnormalities emerged more prominently at the day 14 mark. These findings suggest a greater contribution of these pathways to the chronic muscle weakness observed in post-sepsis conditions, as identified by the key driver feature in gProfiler.

In our analysis, genes altered on both day 4 and day 14 followed one of four distinct patterns: consistent downregulation (n = 72), consistent upregulation (n = 55), a shift from up- to down-regulation (n = 67), or a shift from down- to up-regulation (n = 19). We further subjected genes that exhibited constant up- or down-regulation across these timepoints to a secondary pathway analysis. This was done to identify changes that not only manifested during acute sepsis but also persisted beyond the resolution of the critical phase, as shown in Supplementary Fig. 2.

Predominantly, the pathways identified in this analysis were related to mitochondrial function. GO analysis revealed that metabolic processes and developmental pathways underwent significant alterations throughout the sepsis timeline. Notably, terms related to the tricarboxylic acid (TCA) cycle were consistently downregulated from day 4 to day 14. Highlighting the early onset and persistent nature of mitochondrial-related abnormalities, our findings show that all 15 mitochondrial encoded genes assessed in this sequencing study were downregulated by day 4. By day 14, 10 of these genes continued to exhibit downregulation, as detailed in Supplementary Fig. 3. These transcriptomic changes in mitochondrial genes are likely early events that accumulate, potentially leading to proteomic alterations later in the course of sepsis.

Pathways relating to atrophy were only present during acute sepsis and resolved by day 14. We confirmed atrophy resolution by completing cross-sectional analyses (CSA) of individual muscle fibers of TA muscles using MyoVision software. Significant atrophy was seen on day 4 (Fig. 2D), which corresponded with the overrepresented atrophy pathways. Just as those pathways resolved by day 14, CSA on day 14 was no different than pre-sepsis CSA (day 0).

To evaluate if limited gene expression translated to decreased protein levels, we completed analyses to determine the levels of mitochondrial respiratory chain complexes I-V utilizing Western blotting (Fig. 2E). Through this, we determined that mitochondrial respiratory chain protein levels also decrease progressively after sepsis. Complexes I, II, and V demonstrated consistent decreases in protein expression from day 0 to 4 and again from day 4 to 14. Complex III, responsible for electron transport from ubiquinol to cytochrome c for complex IV’s subsequent use, did not demonstrate any significant differences following sepsis. Protein markers for Complex IV indicated that levels increased from day 4 to 14, but these levels were still significantly lower than day 0 controls. Together, these data indicate that progressive decreases in mitochondrial respiratory chain protein markers mirror the transcriptional changes.

Further, in situ histochemical staining was performed to examine whether the activities of these essential mitochondrial enzymes were also decreased in the skeletal muscle of sepsis survivors.

Through NADH (nicotinamide adenine dinucleotide) dehydrogenase and SDH (succinate dehydrogenase) enzyme staining for complexes I and II activity, respectively, we determined that activity decreased progressively after sepsis (Fig. 2F). NADH activity had no significant decreases from day 0 to day 4 but saw a 20% decrease in staining intensity by day 14. Similarly, SDH activity decreased 8% from day 0 to day 4 and another 20% by day 14, totaling nearly a 30% decrease in SDH activity after sepsis recovery.

Having previously reported significant mitochondrial morphological damage occurring by day 14 (Owen et al. 2019), we completed additional electron microscopy analyses to evaluate the timing of this morphological damage. We found that mitochondrial morphology alters modestly by day 4, and abnormalities accumulate progressively thereafter, with significant damage evident by day 14. Quantification of transcription electron microscopy images indicated that ~ 40% of non-sepsis control animal mitochondria had normal morphology, while septic animals at day 4 (acute illness phase) had only 16.8% normal mitochondria and sepsis survivors at day 14 had even fewer normal organelles (7.5% normal mitochondria) (Fig. 2G).

In summary, our study reveals a progressive accumulation of mitochondrial abnormalities during the phases of sepsis. Initially, these abnormalities appear modestly on day 4, during the acute phase of sepsis. However, they intensify by day 14, in the chronic phase following sepsis resolution. This progression mirrors the worsening skeletal muscle function observed in sepsis survivors, as illustrated in Fig. 1D–E.

### Overexpression of MnSOD protects against *sepsis*-induced muscle weakness.

To examine whether mitochondrial abnormalities *cause* post-sepsis muscle weakness, we utilized a transgenic strain of mice overexpressing the human-derived gene for manganese superoxide dismutase (MnSOD), a mitochondria-localizing antioxidant enzyme that converts superoxide to hydrogen peroxide to be readily handled by catalase, thereby protecting mitochondria from damage (Yen et al. 1996; Holley et al. 2011; Borrelli et al. 2014; Jang et al. 2009). We first confirmed that MnSOD was overexpressed roughly 2.5-fold in the skeletal muscle of transgenic mice (TG) as compared to the littermate wild-type control mice (WT) (Supplementary Fig. 4A). After sepsis induction, mortality rates, degree of hypothermia, and plasma IL-6 levels, a marker of acute systemic inflammation, were all equivalent in TG and WT mice of both sexes, confirming that MnSOD overexpression does not alter overall sepsis severity (Supplementary Fig. 4B-F). As with our previous experiments, septic animals were administered resuscitation fluids and antibiotics 2× daily for 5 days beginning 12 h after sepsis induction. Mice were then euthanized 21 days after sepsis induction for muscle function testing.

Upon euthanasia of the animals for biochemical and histological analyses, we examined the wet weight of hindlimb muscles to further rule out atrophy as a cause of chronic weakness. There were no differences in wet weight as a result of sepsis or overexpression status in the male or female mice for the TA, extensor digitorum longus (EDL), gastrocnemius (GAS), or soleus muscles (Kingren et al. 2021) (Supplementary Fig. 4G-J).

**Fig. 3:**
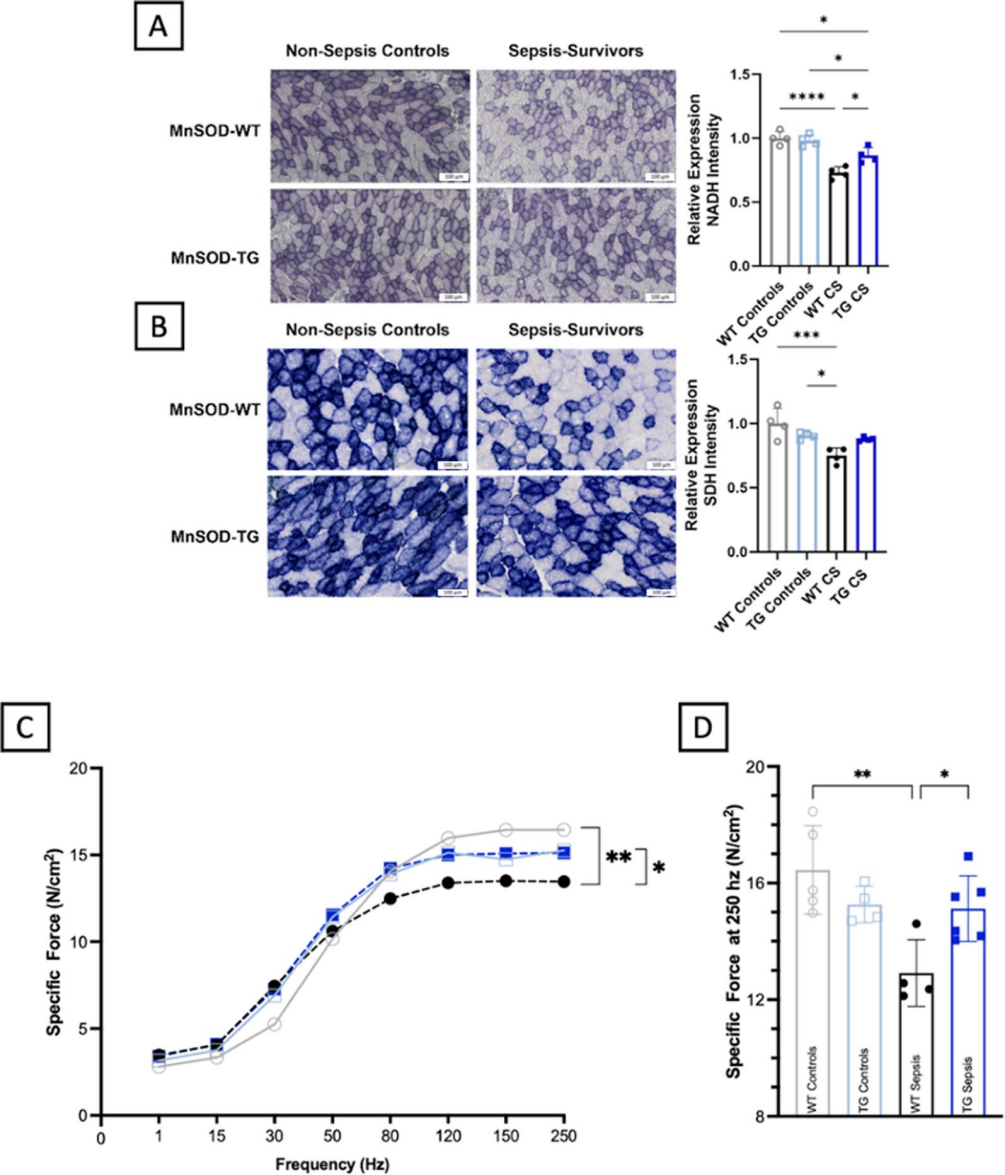
Overexpression of MnSOD protects against sepsis induces mitochondrial and muscular abnormalities. Late middle-aged MnSOD-overexpressing transgenic mice (MnSOD-TG) and littermate wildtype mice (MnSOD-WT) were subjected to experimental sepsis. Sepsis survivor mice were euthanized 21-days post-sepsis induction for in vitro muscle function testing and tissue analyses.** A** NADH staining for complex I activity in TA muscle sections was decreased as a result of sepsis, though MnSOD overexpression provided significant protection when compared to WT sepsis survivors.** B** SDH staining for complex II activity revealed MnSOD overexpression completely protected against typical sepsis reductions in staining intensity. Scale bars (**A** and **B**) represent 100 μm. **C-D** Function was assessed utilizing in vitro function testing 21 days after sepsis induction. MnSOD overexpression completely protected against the muscle weakness found in WT animals, indicating mitochondrial protection prevents chronic muscle weakness. *P < 0.05, **P < 0.01, ***P < 0.001, and ****P < 0.0001

In situ histological staining analyses revealed that mitochondria in the skeletal muscle of TG mice were significantly well protected from sepsis as compared to WT mice. Utilizing NADH and SDH staining for complexes I and II, respectively, we found overexpression of MnSOD prevented the profound sepsis-induced decrease in stain intensity demonstrated in WT sepsis survivors (Fig. 3A–B). While SDH activities in muscle were significantly reduced on day 14 after sepsis in WT mice, such reduction was not seen in TG mice. Though TG mice showed a decrease in NADH activity after sepsis on day 14, this reduction was minimal as compared to the profound decreased activity seen in WT mice (Fig. 3A–B).

We then completed in vitro functional analyses to evaluate if TG mice are protected from sepsis-induced chronic muscle weakness (Fig. 3C–D). Upon subjecting the extensor digitorum longus (EDL) to a force-frequency protocol to determine maximum contraction strength (Owen et al. 2019), significant differences emerged between MnSOD-WT non-sepsis control mice and WT sepsis survivors. These data indicate sepsis imparts a functional deficit of 19.9% that lasts at least three weeks, similar to our previous findings in C57BL/6 mice. However, TG sepsis survivors subjected to the same protocol demonstrated no differences from their TG non-sepsis controls, as well as the WT non-sepsis controls. When compared to the WT sepsis survivors, the TG sepsis survivors were 18.3% stronger. Together, these experiments indicate mitochondrial abnormalities are major drivers of post-sepsis skeletal muscle weakness, as protection of mitochondria throughout sepsis prevented subsequent muscle weakness.

### SS-31 administration after sepsis development prevents chronic weakness

SS-31 (D-Arg-Dmt-Lys-Phe-NH2), a small mitochondria-targeting synthetic tetrapeptide also known as Elamipretide^™^, Bendavia^™^, and MTP-131, selectively targets and concentrates roughly 5000-fold in the inner mitochondrial membrane through electrostatic and hydrophobic interactions (Whitson et al. 2020; Szeto 2014; Liu et al. 2014; Machiraju et al. 2019; Birk et al. 2014; Mitchell et al. 2020; Chavez et al. 2020). The dimethyl tyrosine residues within the peptide provide antioxidant features, and the peptide’s ability to bind to cardiolipin in the inner mitochondrial membrane modulates the opening of the mitochondrial permeability transition pore, thereby preventing cardiolipin’s translocation and peroxidation (Birk et al. 2014; Mitchell et al. 2020; Chavez et al. 2020). As such, SS-31 can confer structural protection, promote ATP synthesis, and decrease electron leakage to limit mitochondrial abnormalities (Birk et al. 2014; Mitchell et al. 2020; Chavez et al. 2020; Sturgeon, et al. 1998). It can also bind to subunits in the electron transport chain protein complexes, further stabilizing and protecting mitochondrial structure and function (Mitchell et al. 2020; Chavez et al. 2020).

Recognizing these advantages, we applied the pharmacological treatment post-sepsis onset to assess its potential in mitigating post-sepsis skeletal muscle weakness and associated mitochondrial abnormalities. We initiated the experiment by administering a typically lethal dose of cecal slurry to mice. This was followed by our established late-stage resuscitation protocol, which includes antibiotics and either a control saline solution or SS-31-enriched saline (0.7 mL saline with 10 mg/kg SS-31, as previously described (Tarantini et al. 2018)). Treatment was administered twice daily for the first five days, then once daily up to day 10 post-sepsis onset.

**Fig. 4:**
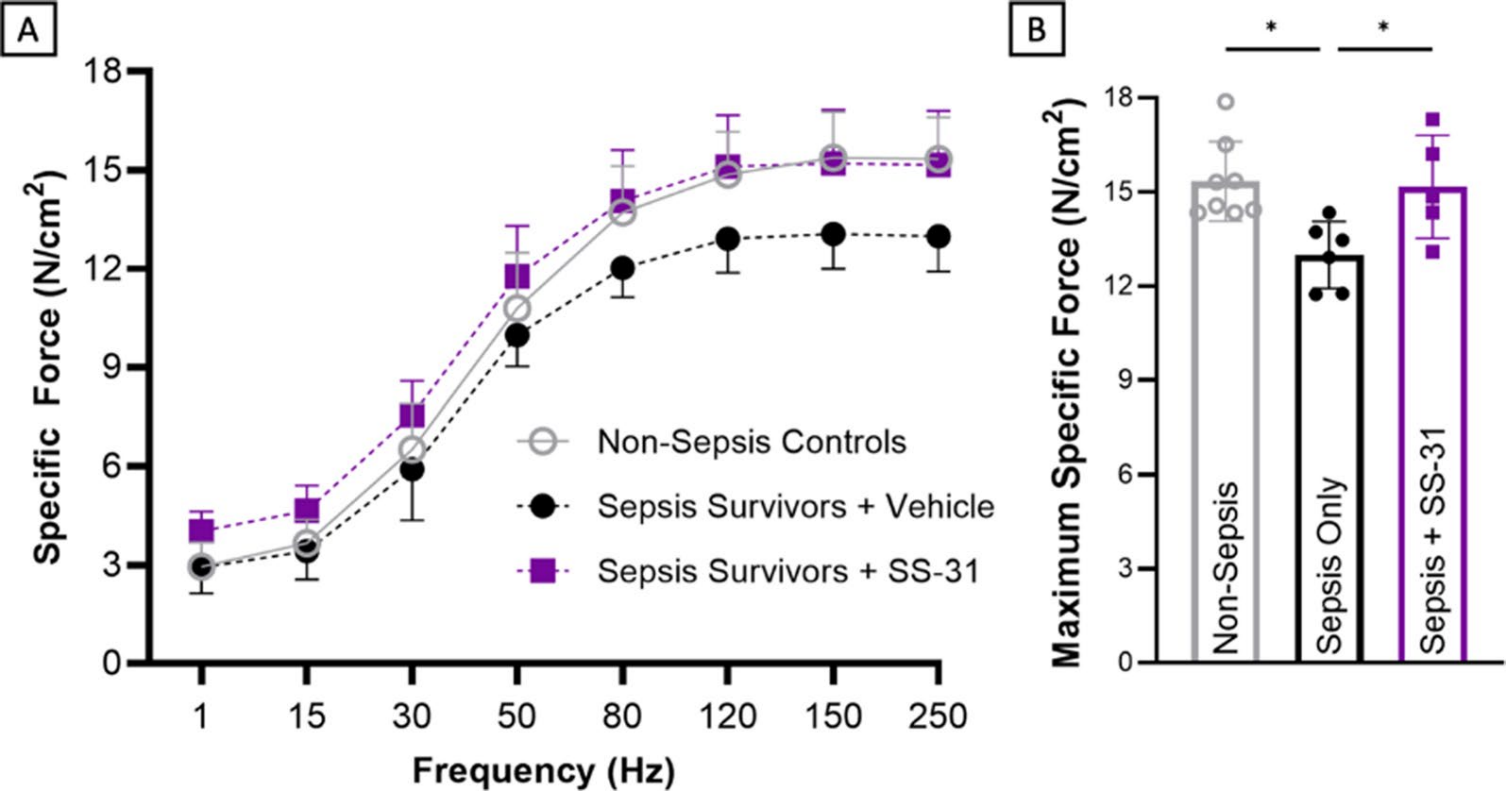
SS-31 administration after sepsis development prevents post-sepsis muscle weakness. Following the development of severe sepsis, mice repeatedly received SS-31 or control vehicle in resuscitation saline starting from 12 h after sepsis induction. In vitro function testing of the EDL on day 21 post-sepsis induction demonstrated that SS-31 protects against muscle weakness development as indicated in **(A)** and **(B)**. *P < 0.05

We completed in vitro function testing on day 21 after sepsis induction to evaluate if the mitochondrial protection conferred by SS-31 led to improved function in sepsis survivors. Mice treated with SS-31 did not demonstrate post-sepsis skeletal muscle weakness—they had no differences as compared to non-sepsis control mice (Fig. 4). Meanwhile, vehicle-treated sepsis-surviving mice suffered 15.3% decreases in maximum specific force. Thus, it is clear SS-31 treatment during acute sepsis prevented the later development of post-sepsis skeletal muscle weakness, evidenced by SS-31 treated mice being 14.8% stronger than their vehicle-treated sepsis counterparts and having no difference from their non-sepsis controls.

### Pharmacological intervention prevents mitochondrial abnormalities

Since SS-31 protected against skeletal muscle weakness, we subjected a second cohort of mice to experimental sepsis to evaluate SS-31’s effects on sepsis-induced mitochondrial abnormalities. We first evaluated how SS-31 administration impacted overall sepsis severity. There were no differences in the survival rate of SS-31-treated and vehicle-treated animals subjected to sepsis (Supplementary Fig. 5A). Plasma IL-6 level at 6-h, an indicator of severe infection, was measured to confirm there were no major differences before drug/vehicle administration (Supplementary Fig. 5E). There were no differences in drug vs vehicle-treated animals in terms of acute hypothermia or prolonged post-sepsis body weight loss, indicating equivalent sepsis severity (Supplementary Fig. 5B, C). Additionally, splenomegaly, an indicator of severe infection, was elevated only in sepsis survivors, with no differences as a result of SS-31 treatment (Supplementary Fig. 5D). Indicating pharmacological intervention had little impact on overall muscle size, there were no differences in post-sepsis EDL wet tissue weight (Supplementary Fig. 5F). As such, we concluded that SS-31 intervention after severe sepsis development does not alter the severity of the initial sepsis event.

**Fig. 5:**
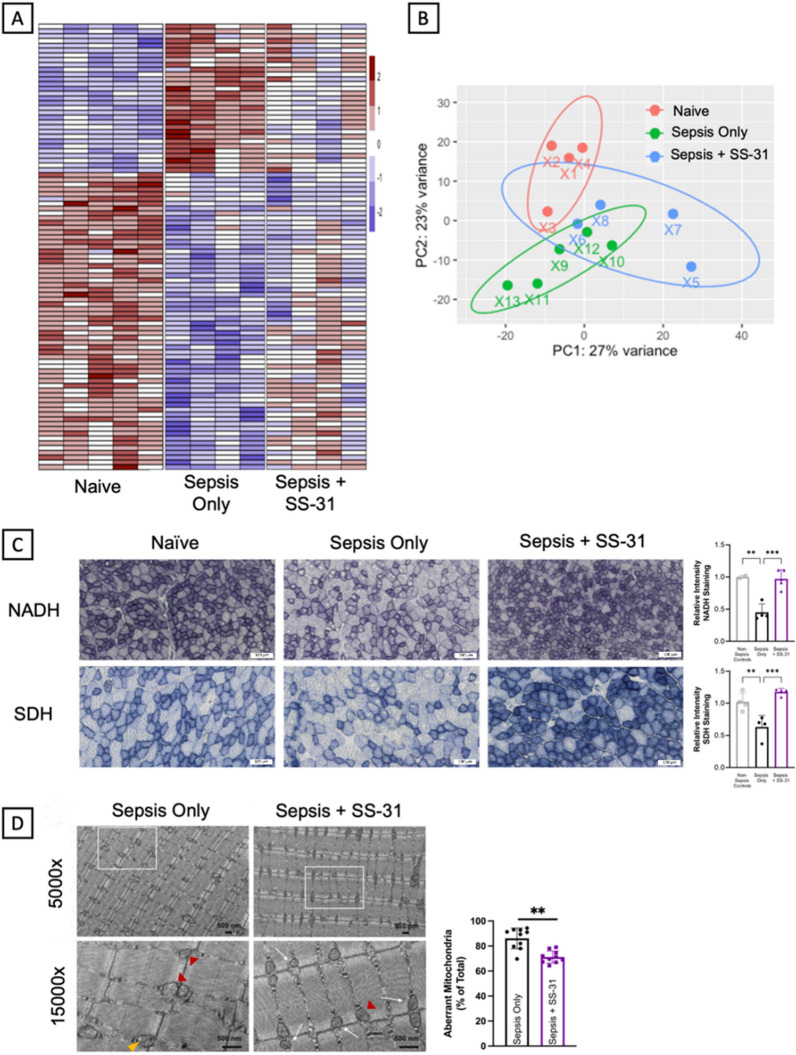
SS-31 administration after sepsis development prevents mitochondrial abnormalities from occurring. **A** Heatmap of DEGs reveal the patterns of SS-31-treated sepsis survivors shift towards naïve controls from vehicle-treated sepsis survivors. **B** PCA indicates clear differences between non-sepsis controls (red) and sepsis survivors, while SS-31 treated sepsis survivors (blue) cluster in between the two other groups. **C** NADH and SDH activity staining in TA muscle sections indicates SS-31 protects against sepsis-induced decreases as shown in the representative images and quantification. Scale bars represent 100 μm. **D** TEM analyses to assess mitochondrial morphology in EDL muscle sections. Representative images of vehicle- and SS-31- treated sepsis survivors (n = 3 each) are shown. Yellow and red arrow heads indicate examples of mitochondria with ruptured mitochondrial membranes and organelles with disrupted cristae/centralization into a vacuole-like structure, respectively. White squares in 5,000x images indicate the area of 15,000x images. Black scale bars indicate 500 nm. There are clear trends indicating that SS-31 confers protection against sepsis induced mitochondrial damage. **P < 0.01 and ***P < 0.001

After euthanizing the mice 28 days post-sepsis induction, we collected TA muscle samples for bulk mRNA sequencing to examine the transcriptomic changes effected by SS-31 treatment. Our analysis revealed distinct clustering of the samples based on whether the mice had undergone sepsis and whether they had received SS-31 treatment. Notably, the SS-31 treated samples occupied an intermediate position in the clustering, situated between the non-sepsis controls and the vehicle-treated sepsis survivors, as depicted in Fig. 5B. Heatmap analysis further highlighted that the transcriptomic profile of SS-31 treated samples more closely resembled that of naïve animals, in contrast to the vehicle-treated sepsis controls (Fig. 5A). This observation was underlined by pathway analysis, which revealed notably fewer mitochondrial alteration-related terms in the SS-31 group. In contrast, vehicle-treated sepsis survivors showed significant changes in pathways related to mitochondrial function, as detailed in Supplementary Fig. 6 and Supplementary Table 1.

When completing histological analyses, we found that SS-31 administration during acute sepsis completely protected against decreases in enzyme activity as marked by in situ staining using NADH and SDH (Fig. 5C). While vehicle-treated animals demonstrated clear deficits in NADH and SDH staining intensity, SS-31 treated sepsis survivors demonstrated no differences from their non-sepsis controls.

During euthanasia, we collected a small portion of the EDL to complete TEM analysis (Fig. 5D). Upon quantifying at least 250 mitochondria per sample (n = 3 per group; 10 separate images were captured and analyzed for each individual sample), we found that post-sepsis mitochondrial abnormalities were significantly decreased in SS-31 treated animals. Further, vehicle treated animals still demonstrated lasting changes in mitochondrial morphology at 28 days, indicating sepsis has a lasting effect that is not cleared by standard mitochondrial dynamics. At 28 days post-sepsis, vehicle treated animals demonstrated 86.1% abnormal mitochondria, while SS-31 treated survivors demonstrated significantly fewer abnormal mitochondria (71.23%; Fig. 5D).

## Discussion

Herein we demonstrated skeletal muscle weakness develops progressively after sepsis induction and persists long after atrophy resolution. Further, we evidenced that mitochondrial abnormalities are key contributors to post-sepsis skeletal muscle weakness. Our work is the first to show that mitochondrial protection prevents skeletal muscle weakness after severe sepsis. With severe weakness affecting a vast majority of severe sepsis survivors, (70–100% (Khan et al. 2006; Witt et al. 1991; Tennila et al. 2000)), this work is highly relevant.

Given that sepsis and post-sepsis syndrome complications disproportionately affect individuals 50 years of age and older (Kingren et al. 2021), our study specifically employed late-middle-aged mice (16–17 months old C57BL/6 strain, equivalent of 50–55 years old humans (Flurkey et al. 2007)). These animals were subjected to the clinically relevant cecal slurry (CS) injection model to induce bacterial abdominal sepsis. This was followed by continuous resuscitation, a process that ensured a high survival rate of mice exhibiting long-term post-sepsis skeletal muscle weakness.

Prior research has shown the occurrence of mitochondrial abnormalities during the acute phase of sepsis (Preau et al. 2021; Singer 2014; Nedel et al. 2023; Azevedo 2010; Mantzarlis et al. 2017). Our recent investigations have further extended this understanding, being the first to demonstrate mitochondrial abnormalities with post-sepsis chronic skeletal muscle weakness in a murine sepsis model (Owen et al. 2019). Nevertheless, the exact timeline and mechanism of mitochondrial damage during the sepsis continuum remained elusive.

We found that mitochondrial abnormalities occur progressively following sepsis and underlie progressively increasing skeletal muscle weakness which persists for at least ten weeks. By utilizing an in vivo function testing system examining plantar flexion function in conjunction with a model of sepsis, we were able to test function before, during, and after sepsis. As such, this is the first evidence that chronic post-sepsis skeletal muscle weakness occurs progressively and is worse following the resolution of atrophy caused by inactivity and tissue sparing during acute sepsis. This finding is particularly important, as most research studies have focused on atrophy as the major driver of post-sepsis skeletal muscle weakness (Cao et al. 2021; Nakanishi et al. 2022). Most of these studies do not study long term weakness; rather, they perform experimentation prior to resolution of the acute sepsis event. They have attributed atrophy as the major driver of muscle weakness post-sepsis. We agree that early phase weakness is overwhelmingly driven by muscle atrophy as indicated by our own findings here and published previously. However, emphasized by decreasing function from the acute phase of sepsis (i.e., days ≤ 3–4) to the chronic phase (day 14 and later) despite recovery from acute illness, it is clear that persistent muscle weakness is not due to atrophy alone. Despite resolution of atrophy gene markers by day 14 and no significant differences in skeletal muscle wet weight or muscle fiber cross-sectional area at days 0 and 14, function was significantly reduced. Notably, this weakness does not improve months after sepsis recovery, as indicated by our day 70 post-sepsis function testing. These findings parallel findings in the clinic. Indeed, skeletal muscle function in sepsis surviving patients has shown to have limited relation to atrophy status 6 months after discharge (Santos, et al. 2016). Accordingly, researchers should focus less on acute sepsis muscle wasting and utilize models that enable study of chronic post-sepsis skeletal muscle weakness.

Our assessment of mitochondrial morphological changes via transmission electron microscopy revealed that mitochondrial damage initiates as early as day 4, during the acute phase of sepsis, and continues to deteriorate through day 14. Notably, these morphological abnormalities in the mitochondria do not resolve even after recovery from sepsis, persisting for at least 28 days post-induction. Complementing these findings, histological analyses demonstrated a progressive decrease in the activity of Complex I and Complex II, as evidenced by in situ staining. Our RNA sequencing analysis also indicates that mitochondrial abnormalities develop during acute sepsis and persist long after recovery, with more mitochondria-related pathways being altered at the 14-day timepoint as compared to the day 4 timepoint. Emphasizing alterations in metabolism occur following sepsis, glycolysis related genes are upregulated, while TCA cycle and ETC related genes are downregulated by day 14. Protein markers for Complexes I-V also demonstrated marked post-sepsis decreases, with Complexes I, III, and IV indicating a progressive decline from days 4 to 14. These changes are paired with decreases in genes encoding Complexes I, IV, and V, as well as cytochrome b, involved in the transfer of electrons through complex III. Similar to our sequencing, histological, and proteomic findings, our previous work examining oxygen consumption rates demonstrated that maximal ADP phosphorylation rate (State III) and Complex I-driven electron transport (State V-CI) were lower in sepsis survivors two weeks after sepsis induction as compared to non-sepsis controls (Owen et al. 2019). Together, it appears that mitochondrial abnormalities—both morphological and functional—parallel worsening muscle function after sepsis.

To investigate whether the observed relationship between mitochondrial damage and skeletal muscle weakness in sepsis was causative, we utilized a transgenic mouse strain overexpressing the mitochondria-localizing antioxidant enzyme MnSOD. MnSOD was selected for its known efficacy in protecting mitochondria against oxidative stress and damage (Yen et al. 1996; Holley et al. 2011; Borrelli et al. 2014) without increasing the lifespan of the animal (Jang et al. 2009). We hypothesized that by elevating MnSOD expression, mitochondria would be better protected during the acute phase of sepsis, thereby preventing the onset of subsequent post-sepsis skeletal muscle weakness. The rationale was that increased MnSOD activity would convert more hydroxide radicals to hydrogen peroxide to be readily handled by catalase (Calaf 2014), reducing acute oxidative damage that contributes to both sarcomeric protein damage and initial mitochondrial abnormalities. We posited that these earlier alterations might set off a vicious cycle, where ongoing mitochondrial damage exacerbates skeletal muscle weakness.

Our findings substantiated this hypothesis. Despite undergoing a comparable severity of sepsis, MnSOD-TG sepsis surviving mice showed a marked resistance to post-sepsis skeletal muscle weakness compared to their WT counterparts. When examining in vitro maximum force, the MnSOD-TG mice recovering from sepsis performed similarly to their non-sepsis transgenic counterparts, indicating a protective effect of MnSOD overexpression. This was further corroborated by the observation that, unlike WT sepsis survivors, the MnSOD-TG sepsis survivors did not exhibit alterations in mitochondrial enzyme activity. These results strongly suggest that mitochondrial abnormalities play a causative role in sepsis-induced muscle weakness and highlight potential avenues for therapeutic intervention.

Since endogenous MnSOD antioxidant overexpression proved to protect against muscle weakness following sepsis, post-sepsis mitochondrial damage is likely driven by oxidative stress. Therefore, it was logical to hypothesize that pharmacological antioxidant intervention following sepsis development could limit this debilitating complication following sepsis development. Already being used in phase I and II clinical trials for its promise in treating acute kidney injury, we decided to evaluate the efficacy of SS-31. Since the drug is water-soluble, we added SS-31 into our resuscitation saline fluids for treated mice in a fashion similar to drug-enriched IVs given to ICU patients. The protective effects conferred by SS-31 in sepsis survivors were similar to those seen in the MnSOD-TG mice. There was no weakness effect seen in drug treated sepsis survivors, while vehicle treated survivors suffered significant muscle weakness one-month post sepsis induction. While markers of acute infection (6 h IL-6 levels and chronic splenomegaly) and sepsis (acute hypothermia and long-term body weight loss) indicate little if any differences in sepsis severity between drug- and vehicle- treated sepsis survivors, we are unable completely rule out that SS-31 had no impact on initial sepsis severity without examining markers of organ damage. Further studies at high doses of SS-31 following sepsis development could identify if the mitochondria-targeting drug can serve as a possible sepsis therapeutic that also prevents post-sepsis skeletal muscle weakness.

## Conclusions

**Fig. 6:**
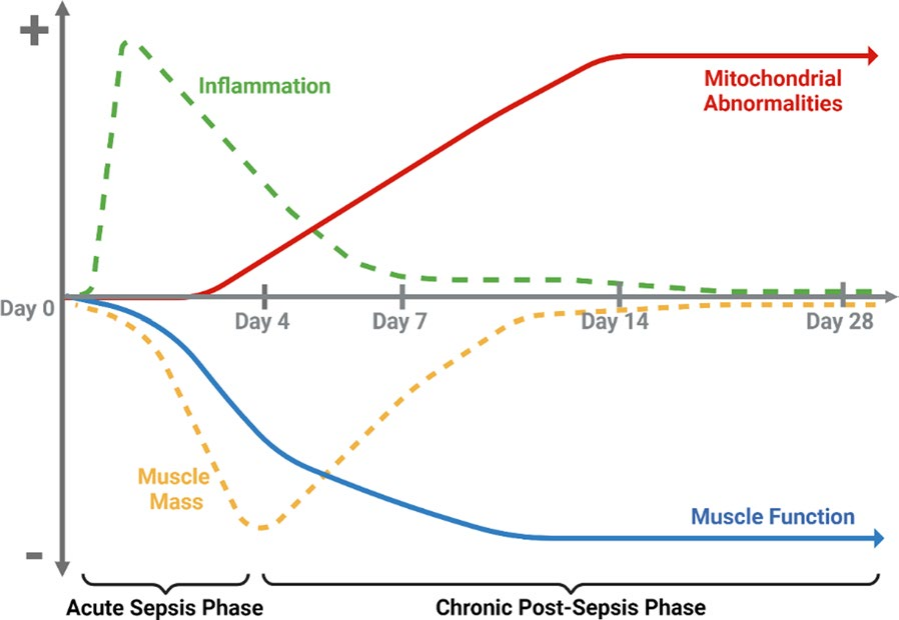
Diagram depicting the development of chronic post-sepsis skeletal muscle weakness. Sepsis induces significant inflammation that resolves early during sepsis pathogenesis. This inflammation is accompanied by acute muscle weakness that mostly resolves within two weeks and progressive muscle weakness. While mitochondrial abnormalities occur early, they accumulate as inflammation and muscle atrophy resolve, leaving altered mitochondrial function and morphology as major causes of chronic skeletal muscle weakness

We summarize the relationship between muscle weakness and mitochondrial abnormalities in Fig. 6. Here, we note that atrophy, while the single most significant contributor to acute sepsis skeletal muscle weakness, mostly resolves within two weeks. Muscle weakness worsens despite atrophy resolution. Mitochondrial abnormalities, however, increase profoundly following acute sepsis, likely a result of free radical production from the initial immune response during sepsis that then worsens in a vicious cycle, leading to further mitochondrial abnormalities and increased muscle weakness. As such, protection of mitochondria from damage during sepsis prevents post-sepsis skeletal muscle weakness, thereby preventing sepsis survivors from suffering from the debilitating condition. These findings are critical as the sepsis incidence rate in western countries has steadily risen nearly 13% annually over the last two decades (Kingren et al. 2021). Alarmingly, this trend is only expected to continue with the increase in aged individuals who are markedly susceptible to sepsis incidence, mortality, and long-term complications.

## Supplementary Information

Additional file 1: **Fig. 1**: Sepsis severity was similar for all mice. (A) Pre-sepsis body weights were similar for mice euthanized at each timepoint for RNA sequencing analyses. Animals were subjected to experimental sepsis and sorted to be euthanized to examine acute and long-term mitochondrial abnormalities. Mice subjected to experimental sepsis (n=6-7 per group) suffered severe disease pathophysiology as emphasized by marked hypothermia (B) and splenomegaly (C). **P < 0.05 and ***P < 0.01. **Fig. 2:** Pathway analysis revealed mitochondrial abnormalities occur progressively. DEGs up- and down- regulated were subjected to GO: Biological Processes analysis, and the results are shown for (A) day 4, (B) day 14, and (C) the DEGs that remained consistently altered. **Fig. 3:** Mitochondrially encoded genes are significantly altered by sepsis. Of the 15 probed mitochondrially encoded genes, 10 remained altered by day 14 as determined by DESeq2 analysis. Normalized reads subjected to DESeq2 analysis were normalized to the day 0 mean for each gene that way differentially expressed in the combined baseline, day 4, and day 14 DESeq2 analysis. All padj values *P* <0.05. **Fig. 4:** MnSOD overexpression does not alter sepsis severity. Mice were subjected to experimental sepsis with late-stage resuscitation to evaluate if MnSOD overexpression alters sepsis pathophysiology. (A) Western blot analysis of MnSOD expression revealed the enzyme is upregulated more than 2.5-fold in TG mice. (B) Survival is not different between TG and WT mice for males or females. (C) Mice exhibited hypothermia for at least 24h after being injected with CS to induce sepsis. There were no differences between WT and TG mouse temperatures. (D) Prior to sepsis induction, there were no differences in animal weights regardless of sepsis or MnSOD grouping. Sepsis survivors suffered significant weight loss, but there were no differences as a result of MnSOD expression status. (E) IL-6 levels were examined after taking tail vein blood at the 6h post-CS injection timepoint. There were no differences between WT and TG animals. (F) Spleen weighs were examined upon euthanasia (day 21) and sepsis survivors demonstrated significant increases, though MnSOD expression status had no effect. The wet weights of hindlimb muscles were taken upon euthanasia for tissue collection. There were no differences in TA (G), EDL (H), gastrocnemius (I), or soleus (J) weights as a result of sepsis or expression status. Male mice are shown on the left, while females are on the right in B-J. K-N were carried out in male animals only. *P < 0.05, **P < 0.01, and ***P < 0.001.** Fig. 5:** SS-31 administration did not alter sepsis severity. Mice treated with either SS-31 or control vehicle showed very similar survival rate (A), Acute hypothermia (B), prolonged weight loss at 3 weeks (C), splenomegaly (D), plasma IL-6 levels at 6-hour after sepsis induction (E), and EDL wet tissue weight (F). These data indicate that SS-31 did not alter overall sepsis severity in mice. *P < 0.05, **P < 0.01, and ****P < 0.0001. **Fig. 6:** SS-31 protects against mitochondrial transcriptomic changes that occur during sepsis. DEGs from vehicle treated sepsis survivors (A) were subjected to GO: Cellular Component (GO:CC) analysis whereupon multiple pathways pertained to mitochondria. However, SS-31 treated sepsis survivors (B) did not demonstrate any GO:CC pathways relating to mitochondria indicating mitochondria protection by SS-31. **Table 1:** Vehicle- and SS-31- treated sepsis survivor DEGs following euthanasia and TA hindlimb muscle collection for bulk mRNA sequencing analysis.

## Data Availability

No datasets were generated or analysed during the current study.

## Abbreviations

ATP: Adenosine triphosphate
CS: Cecal slurry
CSA: Cross sectional area
DEG: Differentially expressed gene
EDL: Extensor Digitorum longus
ETC: Electron transport chain
GAS: Gastrocnemius
GO:BF: Go: Biological Function
GO:CC: Gene ontology: Cellular Component
GO:MF: Gene ontology: Molecular Function
ICU: Intensive care unit
*i.p.*: Intraperitoneal Injection
Lo: Optimal length
MnSOD: Manganese superoxide dismutase
MnSOD-WT: Littermate wild-type control mice for MnSOD-TG mice
MnSOD-TG: Transgenic mice overexpressing MnSOD
NADH: Nicotinamide adenine dinucleotide
*s.c.*: Subcutaneous injection
SDH: Succinate dehydrogenase
TA: Tibialis anterior
TCA: Tricarboxylic acid
TEM: Transmission electron microscopy
PCSA: Physiological cross-sectional area
