# Supplementary material for: Post-sepsis chronic muscle weakness can be prevented by pharmacological protection of mitochondria
Source: Mol Med. 2024 Nov 19;30:221. doi: 10.1186/s10020-024-00982-w (PMC11577827; doi:10.1186/s10020-024-00982-w)
Supplement: Supplementary file 1 — Additional file 1: Fig. 1: Sepsis severity was similar for all mice. (A) Pre-sepsis body weights were similar for mice euthanized at each timepoint for RNA sequencing analyses. Animals were subjected to experimental sepsis and sorted to be euthanized to examine acute and long-term mitochondrial abnormalities. Mice subjected to experimental sepsis (n=6-7 per group) suffered severe disease pathophysiology as emphasized by marked hypothermia (B) and splenomegaly (C). **P < 0.05 and ***P < 0.01. Fig. 2: Pathway analysis revealed mitochondrial abnormalities occur progressively. DEGs up- and down- regulated were subjected to GO: Biological Processes analysis, and the results are shown for (A) day 4, (B) day 14, and (C) the DEGs that remained consistently altered. Fig. 3: Mitochondrially encoded genes are significantly altered by sepsis. Of the 15 probed mitochondrially encoded genes, 10 remained altered by day 14 as determined by DESeq2 analysis. Normalized reads subjected to DESeq2 analysis were normalized to the day 0 mean for each gene that way differentially expressed in the combined baseline, day 4, and day 14 DESeq2 analysis. All padj values P <0.05. Fig. 4: MnSOD overexpression does not alter sepsis severity. Mice were subjected to experimental sepsis with late-stage resuscitation to evaluate if MnSOD overexpression alters sepsis pathophysiology. (A) Western blot analysis of MnSOD expression revealed the enzyme is upregulated more than 2.5-fold in TG mice. (B) Survival is not different between TG and WT mice for males or females. (C) Mice exhibited hypothermia for at least 24h after being injected with CS to induce sepsis. There were no differences between WT and TG mouse temperatures. (D) Prior to sepsis induction, there were no differences in animal weights regardless of sepsis or MnSOD grouping. Sepsis survivors suffered significant weight loss, but there were no differences as a result of MnSOD expression status. (E) IL-6 levels were examined after taking tail [file 10020_2024_982_MOESM1_ESM.docx]

**SUPPLEMENTARY FIGURES:**

**
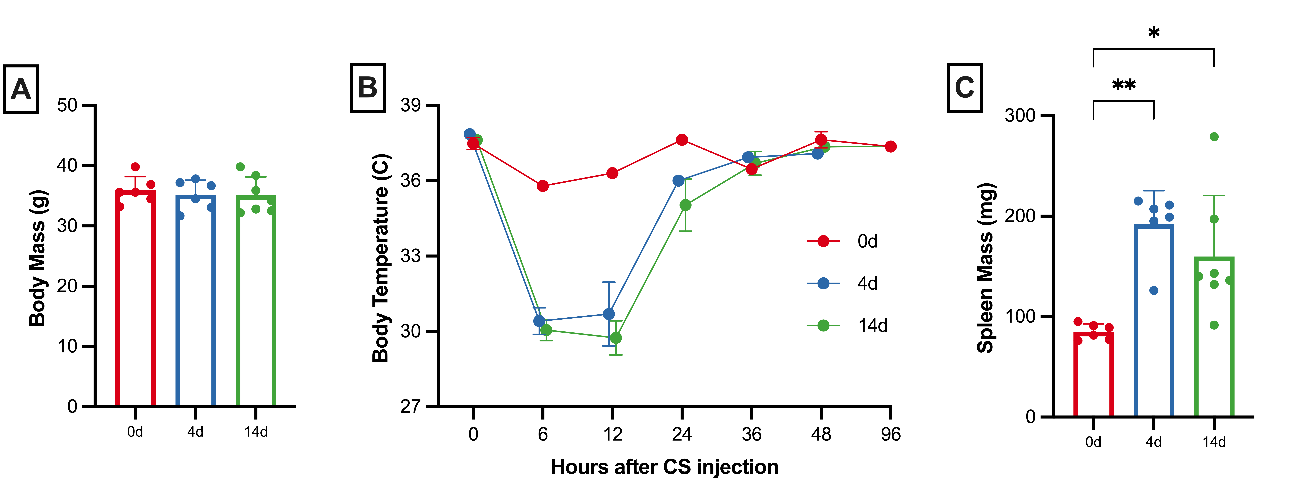
**

**Supplementary Fig. 1: Sepsis severity was similar for all mice. (A)** Pre-sepsis body weights were similar for mice euthanized at each timepoint for RNA sequencing analyses. Animals were subjected to experimental sepsis and sorted to be euthanized to examine acute and long-term mitochondrial abnormalities. Mice subjected to experimental sepsis (n=6-7 per group) suffered severe disease pathophysiology as emphasized by marked hypothermia **(B)** and splenomegaly **(C)**.* *P* < 0.05 and ** *P* < 0.01.


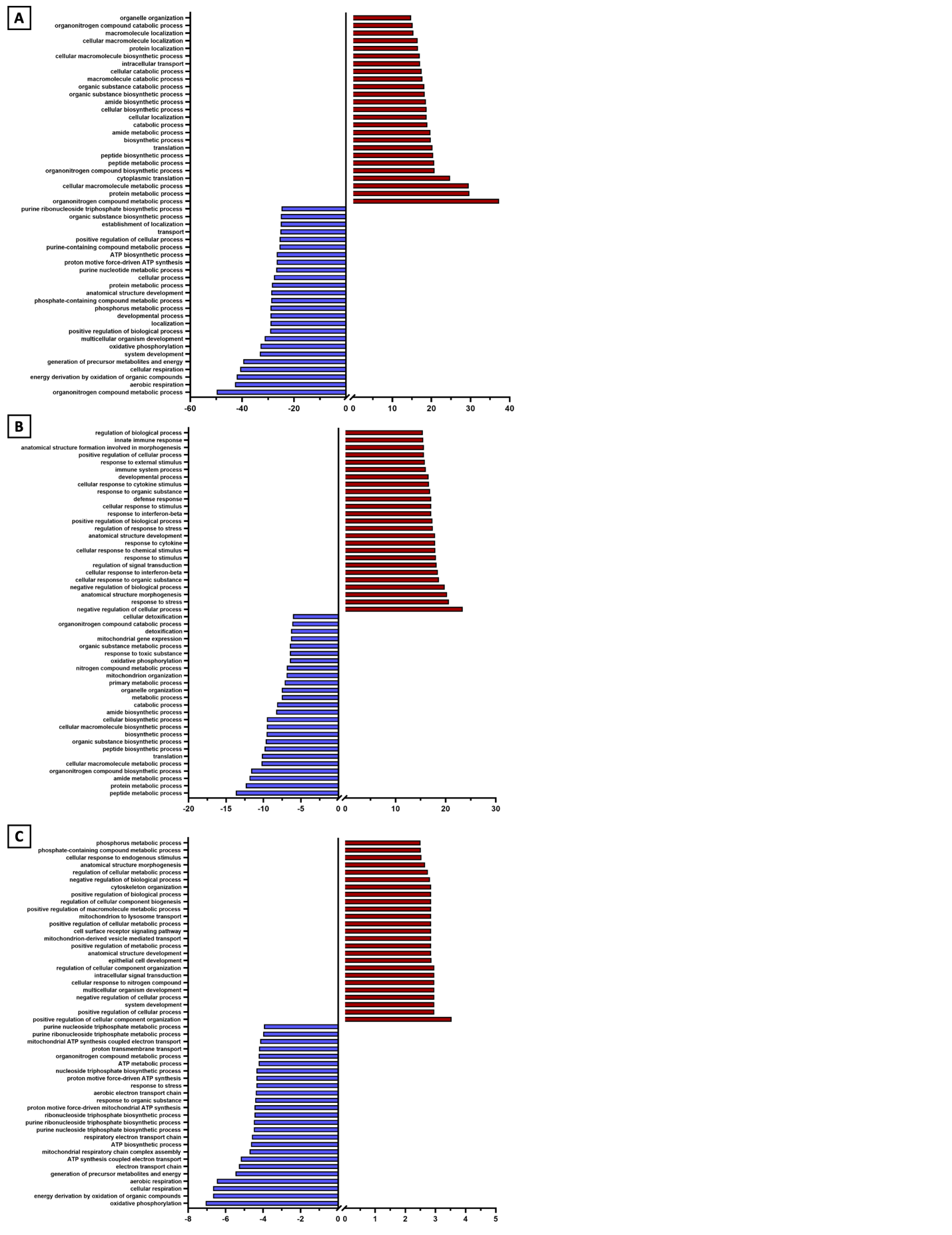


**Supplementary Fig. 2: Pathway analysis revealed mitochondrial abnormalities occur progressively.** DEGs up- and down- regulated were subjected to GO: Biological Processes analysis, and the results are shown for **(A)** day 4, **(B)** day 14, and **(C)** the DEGs that remained consistently altered.


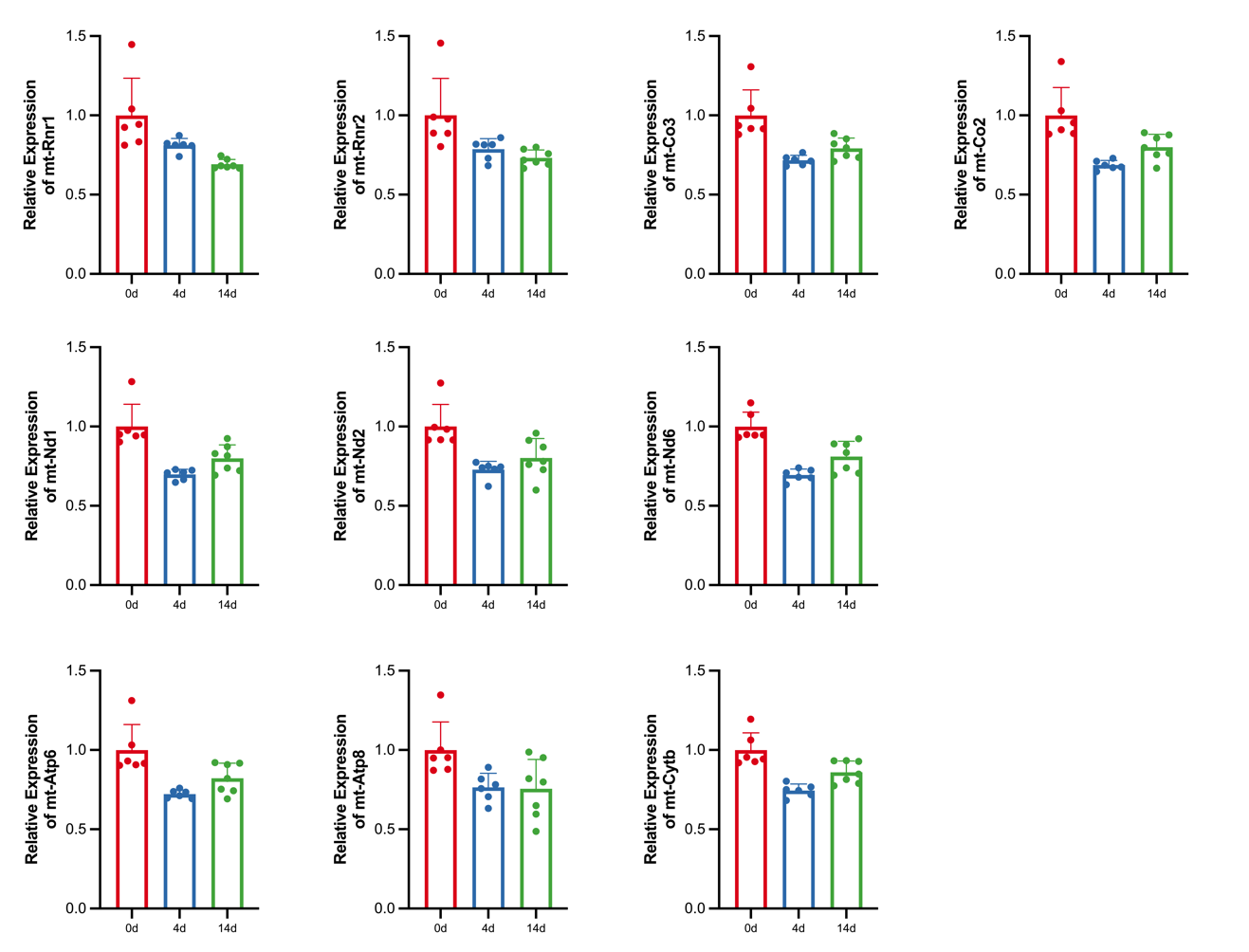


**Supplementary Fig. 3: Mitochondrially encoded genes are significantly altered by sepsis.** Of the 15 probed mitochondrially encoded genes, 10 remained altered by day 14 as determined by DESeq2 analysis. Normalized reads subjected to DESeq2 analysis were normalized to the day 0 mean for each gene that way differentially expressed in the combined baseline, day 4, and day 14 DESeq2 analysis. All padj values *P* <0.05.


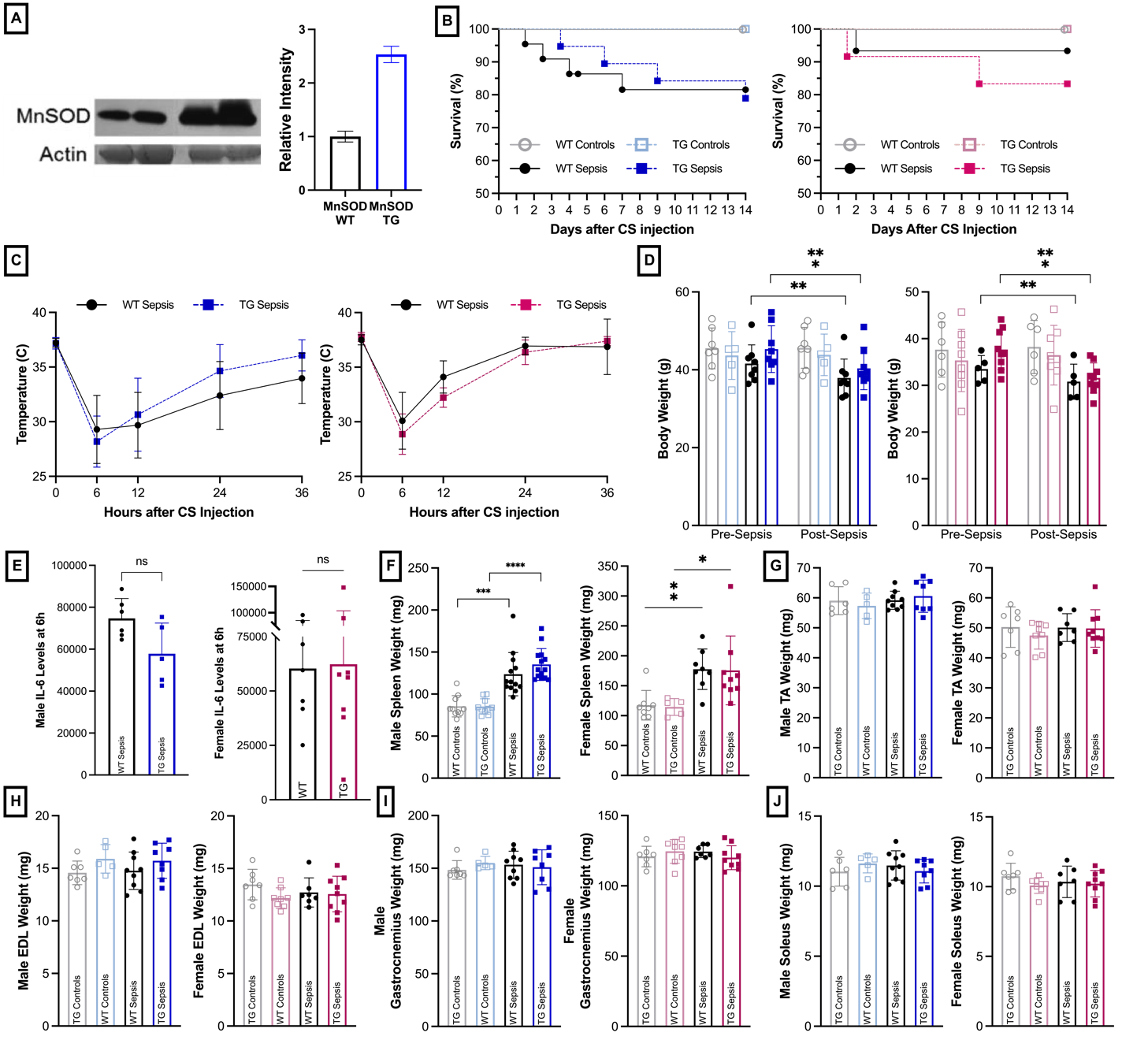


**Supplementary Fig. 4: MnSOD overexpression does not alter sepsis severity.** Mice were subjected to experimental sepsis with late-stage resuscitation to evaluate if MnSOD overexpression alters sepsis pathophysiology. **(A)** Western blot analysis of MnSOD expression revealed the enzyme is upregulated more than 2.5-fold in TG mice. **(B)** Survival is not different between TG and WT mice for males or females. **(C)** Mice exhibited hypothermia for at least 24h after being injected with CS to induce sepsis. There were no differences between WT and TG mouse temperatures. **(D)** Prior to sepsis induction, there were no differences in animal weights regardless of sepsis or MnSOD grouping. Sepsis survivors suffered significant weight loss, but there were no differences as a result of MnSOD expression status. **(E)** IL-6 levels were examined after taking tail vein blood at the 6h post-CS injection timepoint. There were no differences between WT and TG animals. **(F)** Spleen weighs were examined upon euthanasia (day 21) and sepsis survivors demonstrated significant increases, though MnSOD expression status had no effect. The wet weights of hindlimb muscles were taken upon euthanasia for tissue collection. There were no differences in TA **(G)**, EDL **(H)**, gastrocnemius **(I)**, or soleus **(J)** weights as a result of sepsis or expression status. Male mice are shown on the left, while females are on the right in B-J. K-N were carried out in male animals only. **P* < 0.05, ***P* < 0.01, and ****P* < 0.001.


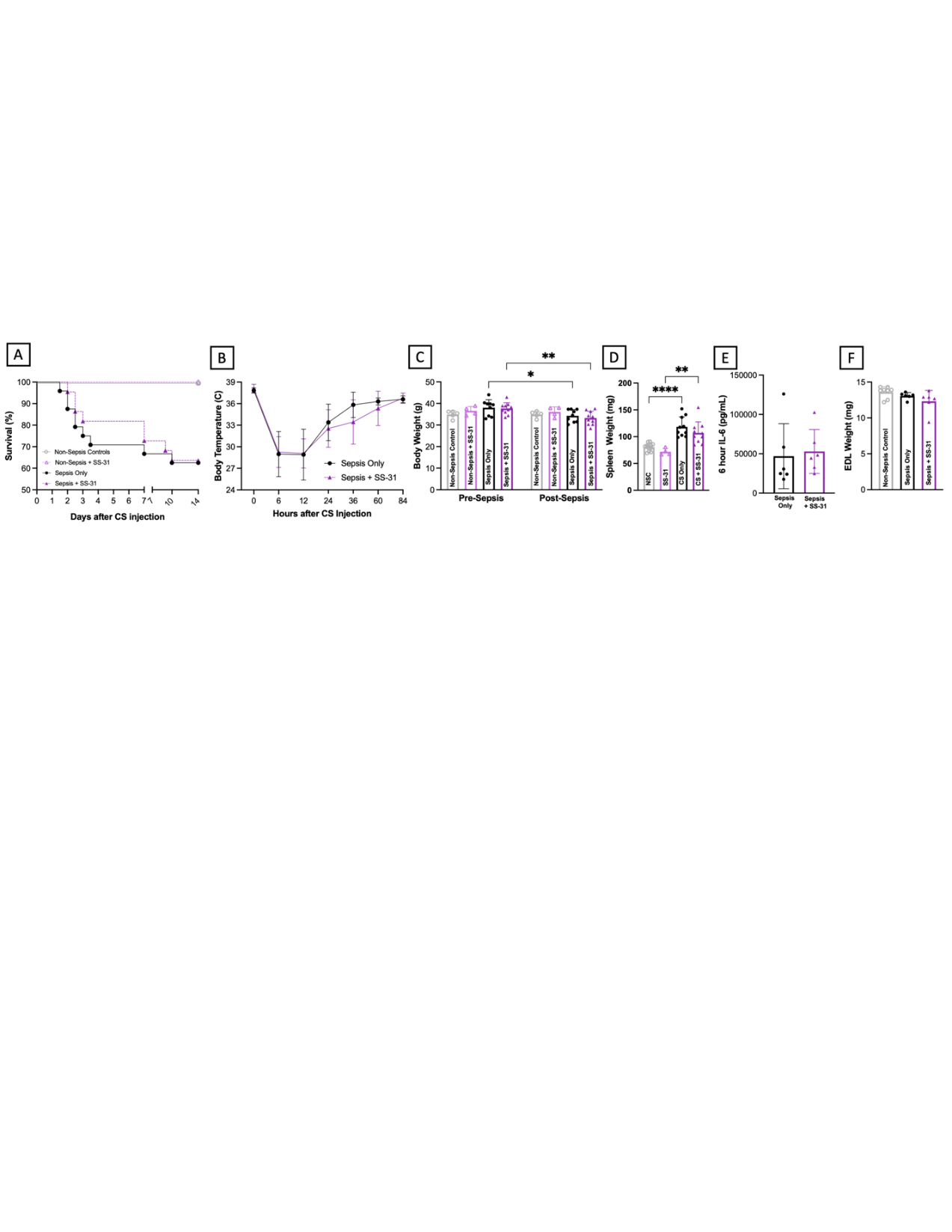


**Supplementary Fig. 5: SS-31 administration did not alter sepsis severity.** Mice treated with either SS-31 or control vehicle showed very similar survival rate **(A),** Acute hypothermia **(B)**, prolonged weight loss at 3 weeks **(C)**, splenomegaly **(D)**, plasma IL-6 levels at 6-hour after sepsis induction **(E)**, and EDL wet tissue weight **(F)**. These data indicate that SS-31 did not alter overall sepsis severity in mice. **P* < 0.05, ***P* < 0.01, and *****P* < 0.0001


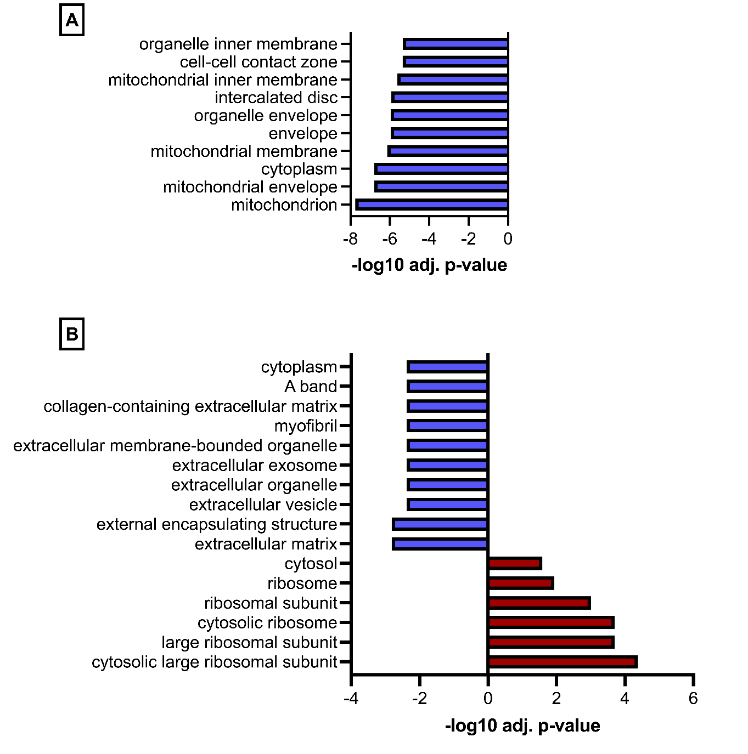


**Supplementary Fig. 6: SS-31 protects against mitochondrial transcriptomic changes that occur during sepsis.** DEGs from vehicle treated sepsis survivors **(A)** were subjected to GO: Cellular Component (GO:CC) analysis whereupon multiple pathways pertained to mitochondria. However, SS-31 treated sepsis survivors **(B)** did not demonstrate any GO:CC pathways relating to mitochondria indicating mitochondria protection by SS-31.


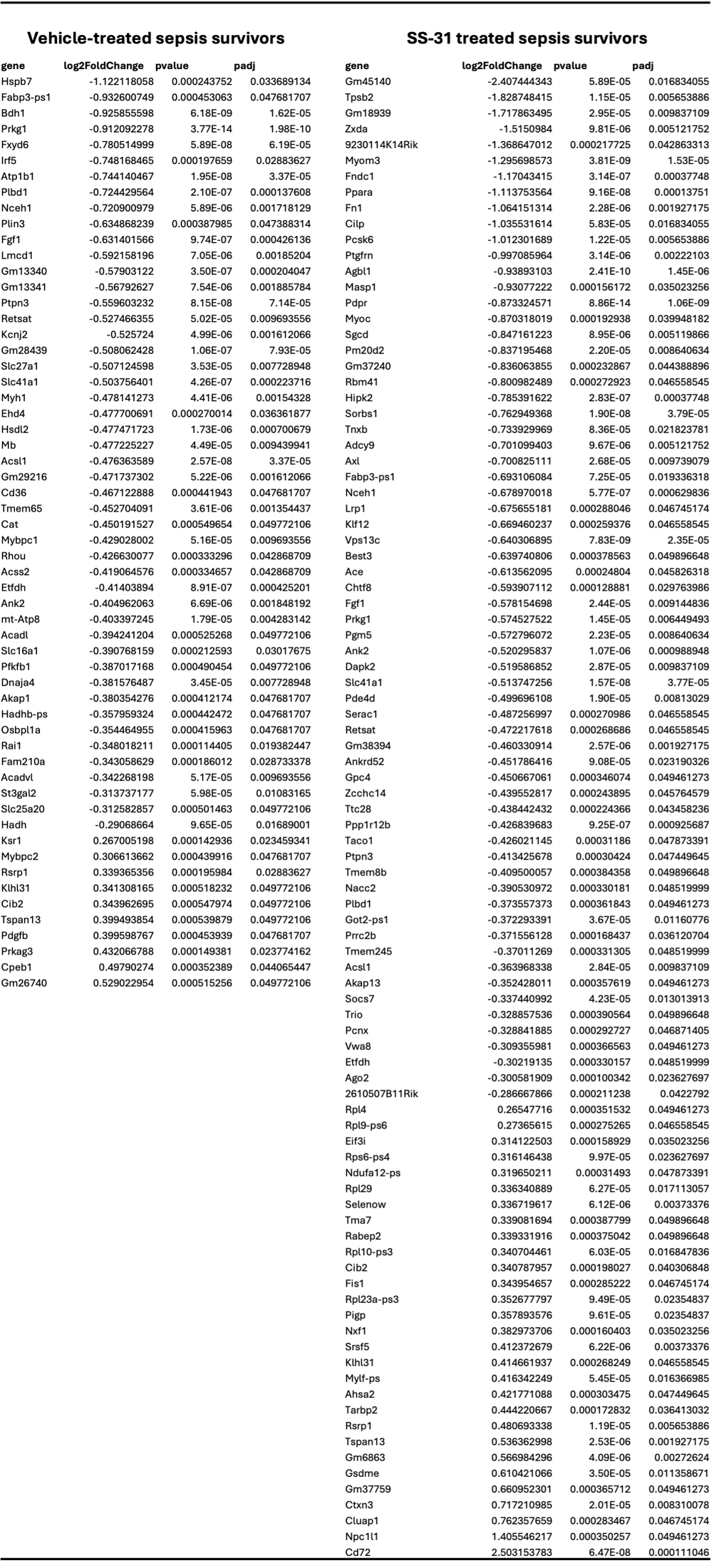


**Supplementary Table 1:** Vehicle- and SS-31- treated sepsis survivor DEGs following euthanasia and TA hindlimb muscle collection for bulk mRNA sequencing analysis.
